# Exploring standing genetic variation for barley leaf rust resistance in Australian breeding panel

**DOI:** 10.1007/s00122-025-05122-4

**Published:** 2026-01-11

**Authors:** Madhav Pandit, Peter Dracatos, Sambasivam Periyannan, Yasmine Lam, Stephanie M. Brunner, Takaaki Honse, Jingyang Tong, Eric Dinglasan, Dini Ganesalingam, David Moody, Silvina Baraibar, Lee Hickey, Samir Alahmad, Hannah Robinson

**Affiliations:** 1 Centre for Crop Science, Queensland Alliance for Agriculture and Food Innovation (QAAFI), The University of Queensland (UQ), Brisbane, QLD Australia; 2 InterGrain Pty Ltd, Perth, WA Australia; 3 Centre for Crop Health, School of Agriculture and Environmental Science, University of Southern Queensland, Toowoomba, QLD Australia; 4 Department of Ecological, Plant and Animal Sciences, School of Agriculture, Biomedicine and Environment (SABE), La Trobe Institute for Sustainable Agriculture and Food (LISAF), Bundoora, VIC Australia; 5 Department of Plant Breeding, Hochschule Geisenheim University, Geisenheim, Germany

## Abstract

**Key message:**

A genotype-by-environment interaction analysis and haplotype mapping approach identifies novel haplo-blocks that can be combined with* Rph20* for enhanced resistance against barley leaf rust.

**Abstract:**

Barley (*Hordeum vulgare* L*.*) production worldwide is threatened by different rust diseases, particularly barley leaf rust (BLR) caused by fungus *Puccinia hordei*. Yet, very limited works have explored BLR resistance mechanism across multiple environments. This study explored genotype-by-environment interactions (GEI) in a BLR disease screening dataset collected over multiple years using a multi-environment trial (MET) analysis followed by iClass method. A haplotype-based approach, using local genomic estimated breeding values (LGEBVs), identified five environmentally stable genomic regions (haplo-blocks: 2HS-b000305, 5HS-b001038, 5HS-b001039, 5HS-b001040 and 5HL-b001125) associated with BLR resistance at adult plant stage. While haplo-block co-locating popular adult plant resistance (APR) gene *Rph20* was validated as a key genomic region to drive stability in resistance across multiple environments, other haplo-blocks with high-effect haplotypes were also reported as prospective novel sources of stability. Notably, environmentally specific haplo-blocks offered insights into GEI-driven resistance mechanisms. The study also highlighted the potential of haplo-block stacking to improve adult plant resistance as genotypes with multiple favorable haplotypes demonstrated a linear relationship with enhanced BLR resistance. These findings hold practical implications for barley breeders, paving the way for more resilient cultivars and advancing breeding methodologies for complex traits like disease resistance.

**Supplementary Information:**

The online version contains supplementary material available at 10.1007/s00122-025-05122-4.

## Introduction

Barley (*Hordeum vulgare* L.) is the fourth largest cereal crop produced globally and mostly used for animal feed, alcoholic beverage production and human consumption. Production of barley is challenged by ongoing changes in the agricultural landscape with increased frequency of biotic stresses, such as fungal diseases. Barley leaf rust (BLR) caused by the fungal pathogen *Puccinia hordei* Otth. is one of the most common and widely distributed rust diseases affecting both wild and cultivated barley (Clifford 1985; Golegaonkar et al. 2009). Typical yield losses of up to 32% in Australia and North America (Park and Karakousis 2002; Ziems et al. 2023) and losses as high as 62% have been reported in susceptible barley cultivars (Cotterill et al. 1992; Hickey et al. 2011). Genetic sources of resistance are the most economical and sustainable disease control and prevention measure for BLR.

To date, 28 *Rph* genes have been designated as the sources of resistance. Two classes of *Rph* genes are recognized: all stage resistance (ASR) genes (*Rph1-19, Rph21, Rph22 and Rph25-28*) and adult plant resistance (APR) genes (*Rph20, Rph23 and Rph24*) (Hickey et al. 2011; Park et al. 2015; Rothwell et al. 2020b; Mehnaz et al. 2021a; Ziems et al. 2023). ASR is a qualitative form of resistance, resulting in hypersensitive response, that is only effective in rust isolates carrying the compatible avirulence (*Avr)* effector proteins, making this type of resistance race-specific and effective throughout the crop growth (Balint-Kurti 2019a, b). However, rust pathogens can evolve to overcome resistance and as such much ASR has been lost over time and virulence is now detected for most known ASR genes (Park et al. 2015; Singh et al. 2021; Mehnaz et al. 2021b). Genotypes carrying APR genes are susceptible at seedling/juvenile growth stage, but plants confer medium to strong level of resistance as they become adult (Rothwell et al. 2018). APR genes are often durable, conferring partial, broad-spectrum resistance against evolving pathotypes and changing environments. However, their quantitative nature and modulation by genotype-by-environment interactions (GEI) complicate breeding efforts. Combining multiple APR and/or ASR genes has been shown to confer stronger and more durable resistance than individual genes with moderate effects. Consequently, breeders are increasingly focusing on stacking diverse resistance genes to achieve stable, broad-spectrum and long-lasting protection (Hickey et al. 2011; Ziems et al. 2017b; Dinglasan et al. 2022).

Beyond gene combinations, the effectiveness of resistance, particularly APR genes, is influenced by BLR pressure and environmental factors, suggesting that GEI may also effect resistance (Singh et al. 2015; Rothwell et al. 2019; Ziems et al. 2023). However, few studies have examined the role of GEI in BLR resistance or its implications for selecting environmentally stable resistance in breeding programs. This gap likely reflects the high cost and time required for phenotypic evaluations across multiple environments. Breeding programs are rich sources of historic phenotypic data, generated as a byproduct of decades of breeding and selection, although unbalanced, can be leveraged using advanced multi-environment trial (MET) analysis and predictive breeding tools. One of the challenges in such datasets is the minimal replication or experimental design used within disease nurseries; however, this can be overcome with the incorporation of a pedigree or a genomic relationship matrix within a MET analysis framework.

Quantitative trait loci (QTL) discovery by association mappings has predominantly been done in diversity panels. However, there remains significant potential to uncover novel sources of resistance within elite breeding panels, where favorable alleles may have accumulated or been inadvertently selected through conventional breeding. To date, this opportunity has largely remained unexplored in case of barley. The fundamental genetic principle underlying association mapping like genome-wide association study (GWAS) assumes that, after multiple generations of recombination, correlations persist only with markers that are tightly linked to the genes governing the trait of interest (Matros et al. 2022). GWAS primarily relies on single nucleotide polymorphisms (SNPs); however, their ability to capture complex relationships between quantitative traits and biallelic SNPs is inherently limited (Wray et al. 2013). This limitation may be mitigated by analyzing haplotype blocks within regions of strong linkage disequilibrium (LD), which are more informative for studying complex traits (Qian et al. 2017). In barley, a predominantly inbreeding species, extensive LD blocks exist across the genome (Rostoks et al. 2006), providing a well-defined haplotype structure (Cockram et al. 2008; Matros et al. 2022). A haplotype-based association mapping has proven effective for identifying genomic loci and candidate genes underlying quantitative traits (Abed and Belzile 2019; Hamazaki and Iwata 2020; Garg 2021; Bhat et al. 2021). Building on this approach, LD-based haplotype analysis using local genomic estimated breeding value (LGEBV) method provides even greater allelic resolution within genomic regions conferring resistance, particularly in diverse breeding panels with strong LD structures (Voss-Fels et al. 2019; Brunner et al. 2024; Tong et al. 2024).

This study represents the first and the largest effort to characterize BLR resistance using a decade of historical breeding data integrated with modern predictive breeding approaches. The proposed strategy demonstrates how historic MET data can be leveraged to assemble large panels with extensive LD blocks, facilitating the detection of genetic effects underlying BLR resistance. Moreover, this framework is broadly applicable to other quantitatively inherited traits with reliable phenotypic records. Specifically, this study aims to: (i) determine genomic regions contributing to BLR resistance that are environmentally stable and environment specific; (ii) dissect the nature of haplotypes for resistance and their existing diversity in the breeding panel; and (iii) explore the potential for stacking desirable haplotypes for improved BLR resistance.

## Materials and methods

### Study material and phenotypic data

This study uses BLR screening datasets of 13,287 genotypes collected over 10 different Australian environments from InterGrain Pty Ltd., an Australian commercial cereal breeding company. The number of BLR observations totaled 16,867 across all environments. To our knowledge, this is the largest panel size of barley genotypes ever assessed to characterize BLR resistance using a breeding panel. The panel consisted of a mixture of advanced breeding lines, along with historical and modern commercially released cultivars, reflecting a long-term breeding objective for economically important traits (e.g., yield, quality, disease resistance, etc.). Genotypic responses to BLR were assessed across 10 distinct environments (location-year combinations) in Australia, utilizing unbalanced randomized disease nurseries grown under field conditions (Supplementary Fig. 1). Typically, commercial cultivars and control/check lines were replicated while breeding test lines were un-replicated within the same nurseries. However, genotypic concurrences were assured across the environments for at least 10–20% of the materials across multiple environments. These nurseries were artificially inoculated with a composite of the prevalent *Puccinia hordei* pathotypes, predominantly *5457P*+*,* for each region where the nursery was assessed. The reaction of genotypes against the disease was scored under field conditions for each hill plot at the post-spike emergence stage (spanning from full spike emergence to the milky ripe phase) using a 1–9 scale, where 1 indicates no observable infection, 9 indicates a full infection or complete susceptibility.

### Phenotypic data analysis

Prior to the main statistical analysis, outlier detection was performed to ensure data quality. While the majority of breeding lines were un-replicated within individual environments, the experimental design included replicated commercial cultivars in each trial. A two-stage outlier identification process was implemented:

*Preliminary check*: Residuals from the replicated cultivars were examined following basic single-site analyses. This allowed for the visual inspection and identification of potential gross errors or anomalies specific to individual plots or measurements within an environment. Data points identified at this stage were treated with caution due to the limited replication.

*Systematic MET-level assessment*: A more comprehensive outlier assessment was conducted using a preliminary MET model fitted to the entire dataset. This model assumed a simpler diagonal variance–covariance structure for the GEI compared to the final factor analytic (FA) model. Using the output from this preliminary MET model, studentized residuals were calculated for each data point, leveraging information across all environments. Observations associated with an absolute studentized residual value exceeding four standard deviations from the mean were flagged as “far outliers” (Tukey 1993; Vo Van-Zivkovic et al. 2025).

Following review, these identified outliers were removed from the dataset. The resulting cleaned phenotypic dataset, comprising 15,481 records across the 10 environments, was used for all subsequent phenotypic analyses.

(i). Single-site analysis

Due to the absence of a structured, replicated field experimental design during disease screening and the unbalanced nature of the dataset, a linear mixed model (LMM) approach was employed to assess the genetic variability in leaf rust scores at each environment. In this model, genotypes were treated as random effects, while residuals accounted for unit-level variation. The linear mixed model can be expressed as follows:

$$y = X_{\tau } + Z_{g} u_{g} + e$$

where ‘*X’* and ‘*Z’* are design matrices associated with fixed (*τ*) and random (*u*_*g*_) effects, respectively, and *e* is the residuals.

The model was iteratively updated to refine the estimates. Residuals were confirmed to be approximately normally distributed, validating the LMM approach (Supplementary information). After re-fitting the model and ensuring no significant outliers remained, additional random effects for replicated observations were incorporated. The genotypes were fitted as random to extract variance components to estimate broad-sense heritability for each environment as per:

$$H^{2} = 1 - A_{tt} /\left( {2\gamma_{v} } \right)$$

where ‘*A*_*tt*_’ is the average standard error of difference and ‘γ_*v*_’ is the genetic variance.

(ii). Multi-environment analysis

Subsequent one-stage MET was conducted using the individual plot data combined across environments to explore the GEI for leaf rust resistance (Smith et al. 2021). First, a diagonal model was fitted where genotype performance in each environment is assumed independent, and each environment has a unique variance and no correlations between environments. In this model, fixed effects include environmental main effects. Due to the nature of the limited experimental designs, genotypic variance was fit as random interaction term within environment in the model to avoid risk of overfitting and/or confounding. The plots of predicted values from this model give insights into how genotypes perform across environments, identify rank changes and detect cross-over interactions, followed by correlation models with assumptions of homogeneous and heterogeneous variation. After determining that the genetic variances and co-variances were not equal between sites, a FA variance structure was fitted to identify the best model for the GEI (Smith et al. 2015). The number of factors was gradually increased until the optimum model was identified utilizing restricted maximum log-likelihood (REML), the Akaike information criterion (AIC) (Akaike 1974), Bayesian information criterion (BIC) (Schwarz 1978) and the total observed genetic variance accounted for by the model. An FA model in the order of three hypothetical factors ${f}_{3}$ was deemed the best model fit, where the genotypic effect and its associated variance were calculated using the following equation:

$$u_{g} = \left( {\lambda_{1} \otimes I_{m} } \right)f_{1} + \left( {\lambda_{2} \otimes I_{m} } \right)f_{2} + \left( {\lambda_{3} \otimes I_{m} } \right)f_{3} + \delta$$

where the coefficients ${\lambda }_{i}$ are the known environment loadings, ${I}_{m}$ represents the genetic variance component of the ${u}_{g}$ variance matrix and $\delta$ is the vector residuals (Smith et al. 2004).

On the final selected FA(k) model, the ‘interaction class (iClass)’ method was used to cluster the similar environments based on rotated loads of each factor from the model. The cross-over GEI within each iClass (cluster) is expected to be the minimal (Smith et al. 2021).

(iii). Phenotypic data analysis for haplotype mapping

To obtain phenotypic values suitable for haplotype mapping while avoiding potential ‘double shrinkage’ from Best Linear Unbiased Predictions (BLUPs), Best Linear Unbiased Estimates (BLUEs) were used. This involved fitting separate linear mixed models where genotype was specified as a fixed effect. Within-iClass BLUEs were generated by fitting a model for each iClass using only data from environments within that cluster, omitting the GEI term. An overall BLUE across all 10 environments was similarly generated by fitting a model to the complete dataset, again treating genotype as fixed and omitting the GEI term. These BLUEs (within-iClass and overall) served as the input phenotypes for the subsequent haplotype analysis. Weighted BLUEs were not applied, as residual variances were modeled directly within environments and the downstream rrBLUP implementation does not support weighting. All the mixed model equations were solved using the restricted maximum likelihood (REML) algorithm. All of the described statistical analyses were performed in R using ASReml-R v4 software (Butler et al. 2017).

A measure of stability for each genotype in terms of the “residual” sums of squares about the regression line was calculated from the first factor of optimal FA model in (ii). The stability measure, root mean squared deviation (RMSD), was utilized and is given by:

$${\mathrm{RMSD}}_{i} = \sqrt {\mathop \sum \limits_{j = 1}^{p} \left( {\tilde{\beta }_{ij} - \hat{\lambda }_{1j} \tilde{f}_{1i} } \right)^{2} /p}$$

where *p* is the number of environments, ${\widetilde{\beta }}_{ij}$ represents the observed or estimated performance measure of the *i*th genotype in the *j*th environment, ${\widehat{\lambda }}_{1j}$ represents the estimated or observed factor loading for the *j*th environment, and ${\widetilde{f}}_{1i}$ represents the factor score or estimated parameter for the* i*th genotype (Smith and Cullis 2018).

### Genotypic data and population structure analysis

Of the breeding panel used for phenotypic analysis, the genotypic data for only 11,819 lines were available for downstream analysis. The lines were genotyped with the Infinium™ wheat–barley 40 K v1.0 BeadChip, returning 12,561 polymorphic SNP markers (Keeble-Gagnère et al. 2021). Missing calls were imputed using Beagle version 5.1 (Browning and Browning 2007). The initial set of markers were curated based on their frequencies, minor allelic frequency (MAF < 0.05) and heterozygous alleles (> 10%) resulting in 5,880 high-quality polymorphic SNP markers to be used in downstream analysis. Genotypes that did not meet the heterozygosity criteria (< 15%) were also removed resulting 11,564 genotypes. Population structure analysis was performed using the ‘SelectionTools’ package (Selection Tools Developers 2022). Genetic distances between lines were computed from the curated marker dataset, generating a genetic dissimilarity matrix based on Rogers’ distance (Rogers 1972). Principal coordinates (PCs) were derived using singular value decomposition, with the first 10 PCs retained and their variance contributions determined from eigenvalues. An optimal number of clusters (K) were decided based on the majority rule using Hubert index in NbClust package (Charrad et al. 2014) in R. Based on this, K-means analysis was applied to structure the population into three distinct clusters. The principal coordinate analysis (PCoA) was drawn as a biplot using “ggplot 2” package in R (Wickham and Wickham 2016).

### Genome-wide haplotype mapping and haplotype stacking analysis

With a final set of 5,880 quality SNP markers, a genome-wide LD threshold of 0.7 between SNPs was used to construct the haplo-blocks separately for each chromosome. A tolerance parameter per block of *t* = 2 was set to account for incorrectly positioned SNP or any possibly biased LD estimates. So, if a flanking SNP did not fulfill the LD threshold, the block was still extended until at least *t* adjacent flanking SNP fulfilled the LD threshold. If more than *t* flanking SNPs had a lower LD than the threshold, the block was completed. This procedure was repeated until all the SNPs were assigned to blocks. SNPs that were not in LD with any other SNP were assigned to individual LD blocks. The range of SNP combinations across a block present in the study panel were called haplotypes. Calculations were performed in the statistical software R using an algorithm implemented in the R package SelectionTools (Selection Tools Developers 2023). Pairwise LD between pairs of markers was visualized using ‘LDheatmap’ (Shin et al. 2006).

Five phenotypic BLUEs generated from overall environments—MET, RMSD and three iClasses were used for five different haplotype study using LGEBV method (Voss-Fels et al. 2019). A ridge-regression best linear unbiased prediction model (rrBLUP) method was applied to determine the effect of every SNP simultaneously (Meuwissen et al. 2001). As per the equation:

$$y = WGu + \varepsilon$$

where *u* is a vector of marker effects, *G* is the genotype matrix, *W* is a matrix relating genotypes to observations (*y*), and *ε* is the error.

These SNP effects were then summed within each haplo-block to quantify the local GEBV of each haplotype, termed as *haplotype effect*. The variance of haplotype effects for each block was determined and plotted as Manhattan plots. Top high-variance haplo-blocks were selected with empirical scaled variance threshold of 0.4 based on the nature of the trait. Haplotypes from each block were deemed favorable if their effect was negative and unfavorable if the effect size was positive.

The top haplo-blocks were further explored for their physical positions on respective chromosomes using start and end marker positions to align them against barley reference assembly, ‘Morex’ v2 (Monat et al. 2019) using Basic Local Alignment Tool (BLAST) on GrainGenes platform (Yao et al. 2022). The physical positions of 22 *Rph *genes were obtained from previous studies (Ziems et al. 2023; Mehnaz et al. 2021a) and some by using BLAST with previously reported linked markers. The haplo-blocks and known *Rph* genes were visualized on respective chromosomes using Mapchart 2.32 software (Voorrips 2002). An ‘UpSet plot’ was developed for top haplo-blocks by using the *UpSetR* package in R environment (Lex et al. 2014).

Haplotype ‘stacking’ was performed to investigate the additivity of multiple favorable haplotypes to improve leaf rust resistance. This was carried out by determining the number of beneficial haplotypes present in each genotype for the subset of top blocks. The average trait value for individuals grouped by number of blocks was then used to explore the effect of multiple haplotypes on the trait. Out of top haplo-blocks, blocks that were co-located across the five sets of LGEBV analysis were identified as environmentally stable blocks. The haplotype effects across the stable blocks were plotted against the PCoA biplot using ‘ggplot2’ package in R to visualize their distribution across the three clusters of the breeding panel.

## Results

### Insights on genetic diversity and population structure

**Fig. 1:**
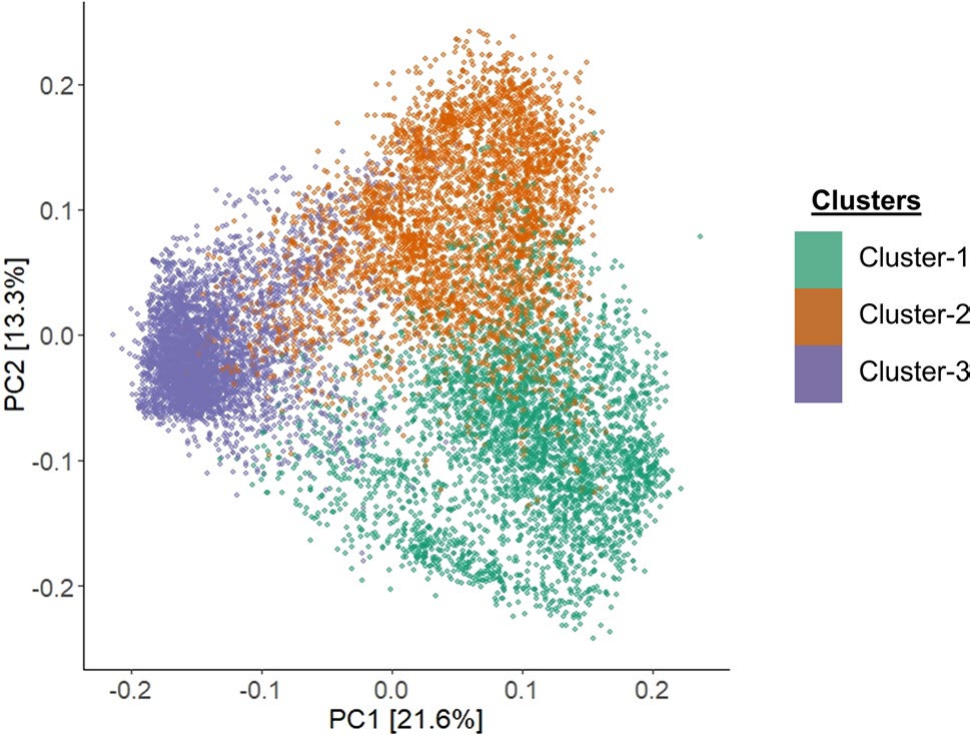
Genetic diversity of the barley breeding panel revealed by principal coordinate analysis (PCoA) performed based on Roger’s distance and calculated using 5,880 SNP markers

A total of 5,880 high-quality SNP markers were used to conduct a population diversity analysis of the barley breeding panel. Principal coordinate analysis (PCoA), based on Roger’s distance, identified three major clusters within the study panel (Fig. 1). The first and second principal components accounted for 21.6% and 13.3% of the genetic variance, respectively. The breeding panel was clustered into three distinct clusters. Collectively, these clusters capture high genetic diversity of barley germplasm available in Australia, originated from different crossing strategies and geographical origins.

### Multi-environment analysis to uncover phenotypic variation for adult plant resistance

Significant variation in BLR scores was observed across 10 environments (Table 1). The highest BLR variance was recorded in environments 2018_ENV1 (2.87), followed closely by 2019_ENV1 (2.86), with corresponding estimated genotypic variances of 7.45 and 7.36, respectively. Conversely, the lowest BLR variance was observed in 2017_ENV1 (1.30), accompanied by the smallest genotypic variance estimate (0.87). Using *diag* model, a high range of genotypic variance (0.87–7.45) across environments was observed. Furthermore, significantly high z-ratios (> 2) observed across each environment indicate that genotypes contribute significantly to the existing phenotypic variability across environments. A medium-to-high heritability (0.52–0.90) was observed for the BLR response. To unravel possible interactions between genotype and environment, a multi-environment trial (MET) analysis was conducted.

**Table 1:** Descriptive statistics of field disease response for barley leaf rust (BLR) across environments (Year_location), (Z-ratio: Genotypic variance/standard error). The full description of the environment has not been revealed due to associated IP and data privacy

Environments	No. of genotypes	BLR mean	BLR median	BLR variance	Genotypic variance	Z-ratio	Heritability (*H*^*2*^)
2016_ENV1	283	5.90	6.20	1.49	1.41	7.03	0.62
2017_ENV1	339	4.01	3.70	1.30	0.87	6.13	0.65
2018_ENV1	495	5.75	5.00	2.87	7.45	14.08	0.90
2018_ENV2	417	4.83	5.00	2.09	3.51	11.76	0.52
2019_ENV1	1256	5.98	5.00	2.86	7.36	22.15	0.71
2020_ENV1	3358	4.09	4.00	1.46	1.32	16.59	0.63
2021_ENV1	4586	3.50	3.00	1.53	1.55	19.86	0.83
2022_ENV1	4138	4.17	4.00	1.68	2.01	23.23	0.85
2022_ENV2	455	4.83	5.00	2.20	3.70	12.70	0.90
2022_ENV3	906	6.87	7.00	1.62	1.80	13.18	0.69

The predicted performances (BLUPs) of each genotype within each environment, calculated from the MET analysis, were plotted as line graphs (Supplementary Fig. 1 and 2) and revealed the presence of the cross-over and rank GEI. Also, from the output of the MET analysis, the FA3 model was deemed the best model fit, explaining overall 82% of the total genetic variance and thus was used for environmental clustering analysis (Table 2). Each of the three factors from FA3 model accounted different levels of variance across the environments with 100% variance accounted for six environments (2020_ENV1, 2021_ENV1, 2016_ ENV1, 2017_ ENV1, 2018_ ENV2 and 2022_ ENV3) (Table 3). The lowest variance was recorded for 2018_ENV1 (57.02%).

**Table 2:** Goodness of fit for multi-environment trial analysis across linear models for barley leaf rust score

Model	AIC	BIC	LogLik	V%
Diag	33,668	33,761	− 16,822	NA
CorV	34,394	34,425	− 17,193	NA
CorH	32,705	32,805	− 16,339	NA
FA1	32,659	32,806	− 16,310	67
FA2	32,655	32,863	− 16,300	74
**FA3**	**32,645**	**32,900**	− **16,289**	**82**
FA4	32,648	32,941	− 16,285	90

Bold represents the selected model based on AIC, BIC and LogLik. Diag: Diagonal model, CorV: Correlation model with homogenous variation, CorH: Correlation model with heterogenous variation, FA: Factor analytic models with one to four factors, Model Parameters (AIC: Akaike information criterion, BIC: Bayesian information criterion, LogLik: REML Log-likelihood and V%: total variance explained

**Table 3:** Summary of variance components, the rotated REML estimates of loadings and iClass designation from the FA3 model fit the GEI term in MET analysis

Environment	Factor 1		Factor 2		Factor 3		Total variance	iClass
	Var	Loading	Var	Loading	Var	Loading		
2020_ENV1	72.78	1.22	22.18	− 0.55	5.04	− 0.25	100.00	PNN (0.61–0.92)
2021_ENV1	47.30	1.05	18.80	− 0.51	33.89	− 0.75	100.00	
2018_ENV1	56.31	2.42	0.70	− 0.4	0.01	− 0.46	57.01	
2017_ENV1	62.15	0.89	35.00	− 0.58	2.84	0.01	100.00	PNP (0.7–0.99)
2022_ENV1	56.00	1.27	0.05	− 0.12	2.65	0.95	58.72	
2016_ENV1	96.17	1.38	1.43	− 0.27	2.40	0.01	100.00	
2018_ENV2	90.49	2.06	2.84	− 0.42	6.66	0.49	100.00	
2022_ENV2	90.41	2.27	1.36	− 0.47	2.52	0.21	94.29	
2019_ENV1	66.73	2.74	15.03	1.65	0.65	− 0.15	82.42	PPN (0.87)
2022_ENV3	81.00	1.48	14.26	0.41	4.73	− 0.05	100.00	

Var: variance accounted for in each environment by each factor in FA3 model; Loading: factor loading and iClasses with ‘P’ and ‘N’ representing positive and negative loadings of the respective factors, respectively. Figures inside parentheses () are the range of genetic correlations between pairs of environments within the respective iClass

**Fig. 2:**
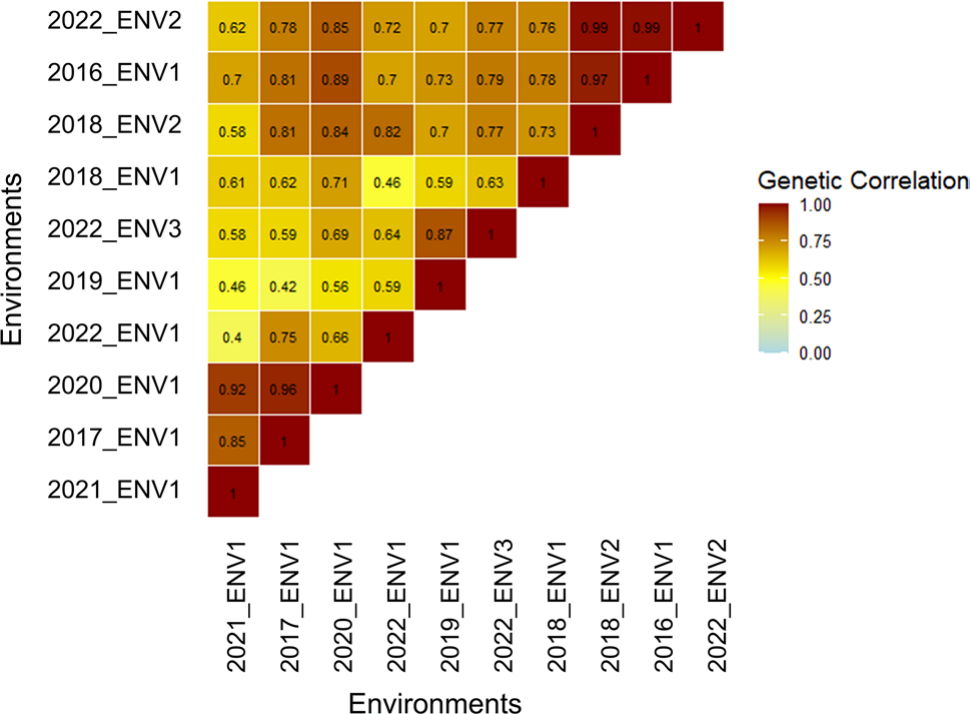
Heatmap of genetic correlations among environments estimated based on the variance–covariance matrix of the FA3 model

The polarity of the loadings for three factors from FA3 model was utilized to identify clusters of environments termed as iClasses (Table 3). Three iClasses (PNN, PNP and PPN) were formed, and respective phenotypic BLUEs for each iClasses and across all environments-MET were estimated. Here, since the genotypes were unbalanced across the environments, each iClass maintained different genotype entries, yet a high level of concurrence was maintained across the iClasses. Medium-to-high (0.61–0.99) genetic correlation between the pairs of environments within an iClass (Fig. 2) suggested the similar environments were grouped into the same clusters. Duncan’s multiple range test (DMRT) was carried out to observe the pairwise comparisons between the mean BLUEs across the MET and the three iClasses, which revealed significant difference in group-wise means signifying importance of carrying out separate haplotype analysis for each group (Supplementary Fig. 3).

### Unveiling haplo-blocks associated with leaf rust resistance across environments

LD among the SNP markers was used for constructing genome-wide haplo-blocks. The study panel exhibited pronounced LD across the seven chromosomes and a slow genome-wide LD decay (Supplementary Fig. 4 and 5). With progressive increment in LD threshold and tolerance values (Supplementary Table 1), a higher LD threshold of 0.7 and tolerance (*t*) of two were used to construct haplo-blocks. This approach resulted in the identification of 2,033 chromosomal regions (haplo-blocks) spanning across the seven chromosomes. The average block size consisted of 2.89 SNPs and 34 haplotypes per block. While 1,281 blocks comprised a single SNP (with positive, negative, or zero effect sizes), the largest block, b001093 on chromosome 5H, included 143 SNPs and 3,749 haplotypes. Importantly, no significant correlation was observed between block size (number of SNPs or haplotypes) and associated block variance (Supplementary Fig. 6).

**Fig. 3:**
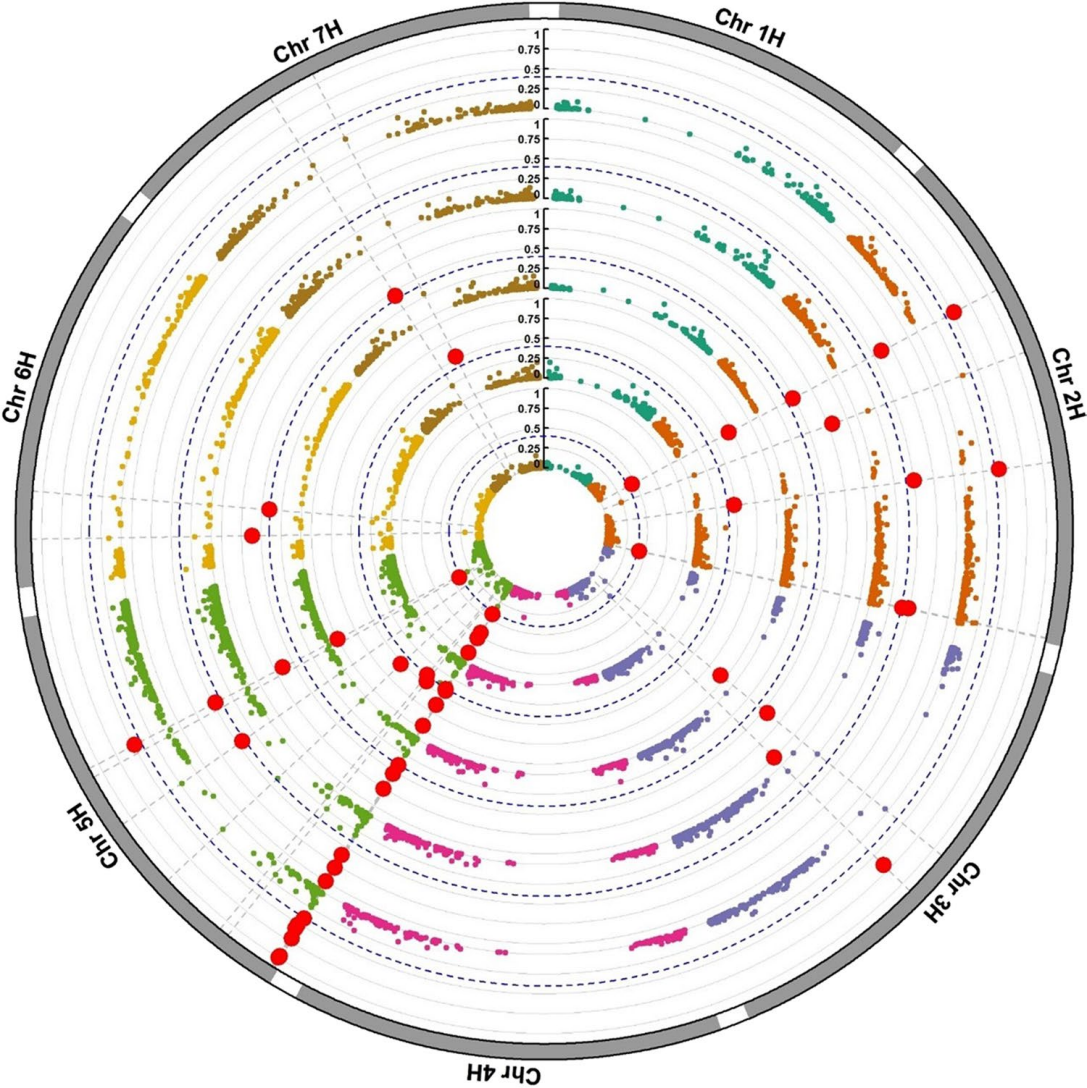
A combined circular Manhattan plot to show haplotype effect variance (*y*-axis) per haplo-blocks across the seven chromosomes (*x*-axis). A threshold scaled variance 0.4 was used as a measure of selecting important haplo-blocks across all analyses associated with the leaf rust resistance. Each red dot displays the top haplo-blocks exceeding the threshold value

Five sets of LGEBV analyses were performed to identify haplo-blocks associated within each environment cluster, as defined by the iClasses, as well as across all environments and for the stability measure-RMSD. Group-wise, a scaled min–max variance threshold of 0.4 was used to identify the important blocks within each haplotype analysis. The analysis identified nine haplo-blocks for the all-environment analysis (MET), eight for iClass PNN, 12 for PNP, 10 for PPN and nine for RMSD (Fig. 3). Across all five analyses, a total of 21 high-variance blocks associated with BLR resistance were deemed important for breeding. The distribution of 21 high-variance haplo-blocks across the seven chromosomes and environmental context are also presented using an UpSet plot (Supplementary Fig. 7; Lex et al. 2014). The plot illustrates the size of each set (e.g., total number of high-variance haplo-blocks identified in each analysis) and as well as common high-variance haplo-blocks detected across analyses. Notably, five LD blocks were consistently detected across all analyses, whereas the iClass-specific haplo-blocks highlight potential environment-specific sources of resistance.

**Fig. 4:**
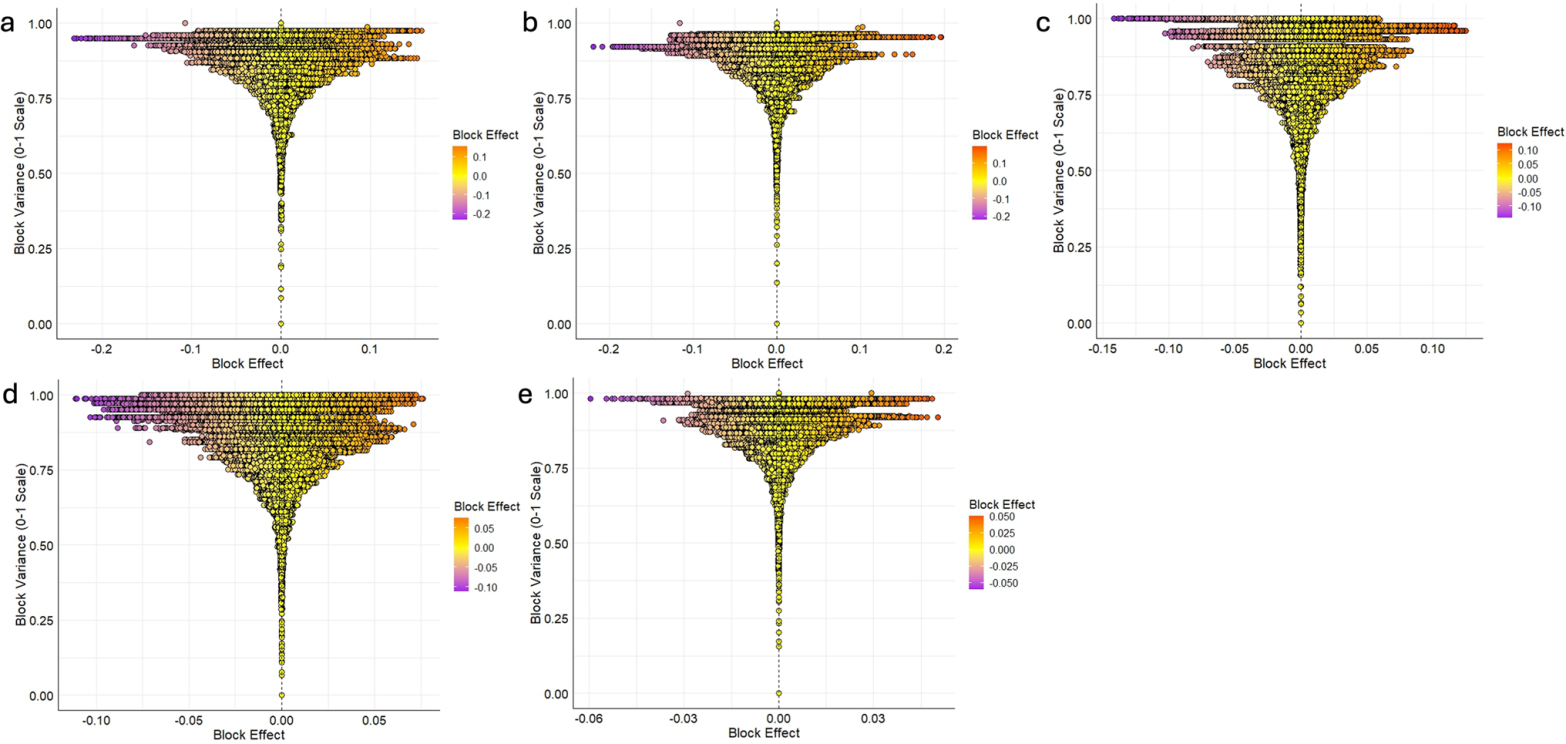
Haplotype effects from 2033 haplo-blocks plotted against respective block variances calculated from five haplotype analyses **a** MET, **b** PNN, **c** PNP, **d** PPN and **e** RMSD. Each horizontal combination of dots is for one specific haplo-block with variation in effect size at the haplotype level. A negative effect haplotype is associated with contributing toward lowering the rust score (i.e., toward resistance)

For the 21 haplo-blocks, their chromosomal positions (start–end, Mbp), SNP counts and number of haplotypes varied across and within iClasses, MET and RMSD analyses (Supplementary Table 2). Notably, block b001039 consistently demonstrated the highest variance across MET, PNN and RMSD. In contrast, blocks b000602 and b001125 exhibited the highest variance in iClasses PPN and PNP, respectively. Five prominent environment-stable haplo-blocks were identified across all five analyses: b000305, b001038, b001039, b001040 and b001125. In addition to the haplo-block variance, the extent of effect contributed by the individual haplotype is important for selecting any haplo-block as favorable. For any genotype, BLR score toward 1 represents the resistance nature of the genotype, and as such, identifying haplotypes contributing negative effects are important. However, the largest effect haplotypes were not always found in the blocks with highest variance. The LGEBV (block effect) and their respective block variances were plotted on the *X* and *Y* axes respectively (Fig. 4). Each horizontally positioned dot represents the existing haplotype present in the panel for a specific haplo-block. Although haplo-block b001039 was the highest variance haplo-block across MET, PNN and PNP, the haplotype with largest effect for resistance was found in b001125. Similarly in case of PPN, the highest variance haplo-block was b00602, but the haplotype with largest effect for BLR resistance was found in b000306.

### Haplo-blocks and their co-location with known Rph genes

**Fig. 5:**
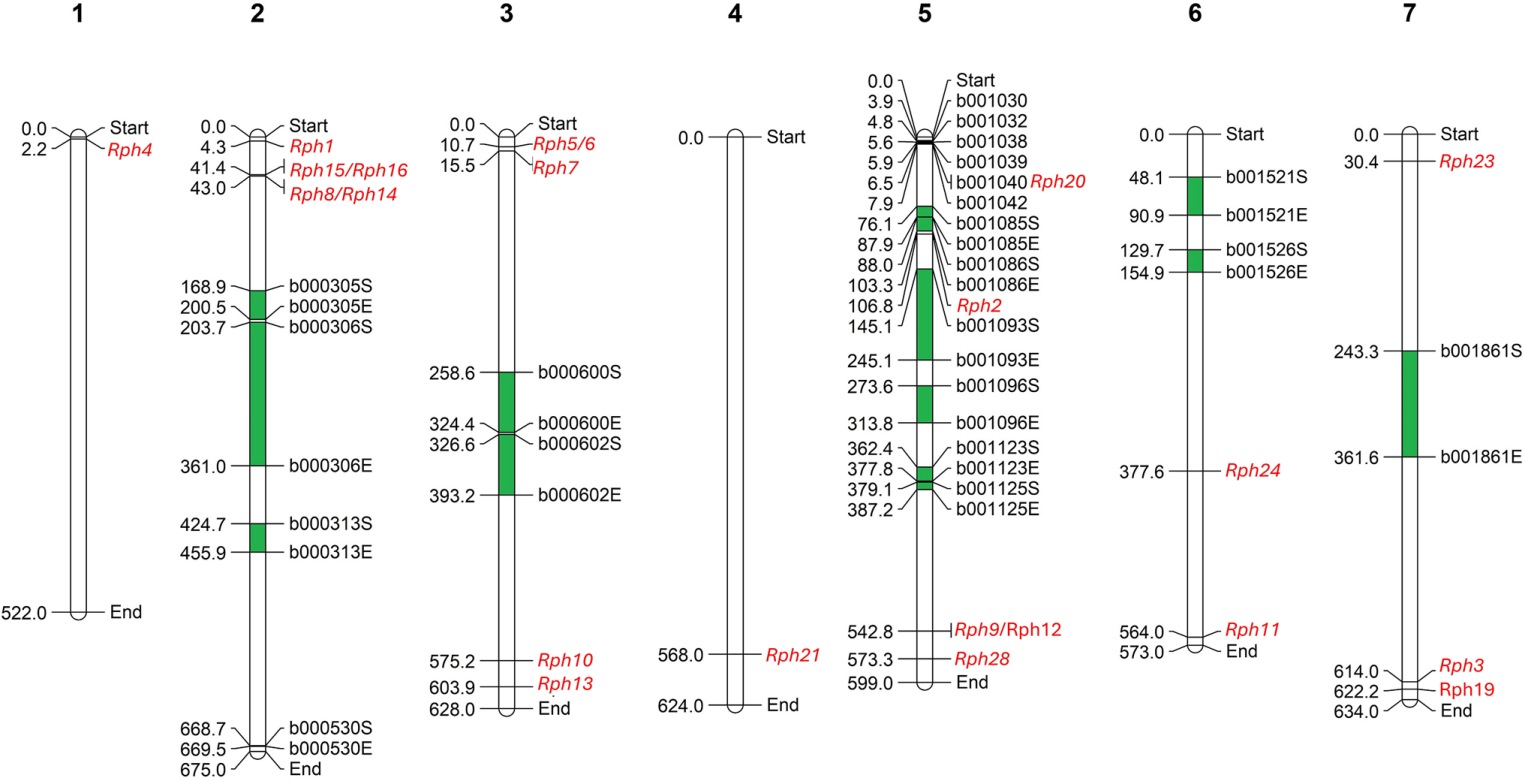
Visualization of 21 haplo-blocks (in green) and known *Rph* genes (in red) across seven chromosomes (1–7), their physical positions (Mbp) based on BLAST results on Morex v2 by TRITEX (2019). The suffixes ‘S’ and ‘E’ after each block names represent start and the end positions of respective haplo-block, respectively

A comprehensive literature review identified known BLR resistance genes (*Rph* genes) and BLAST analysis determined the positions of 22 *Rph* genes and 21 haplo-blocks, which were mapped on Barley Morex V2 genome assembly by TRITEX (Monat et al. 2019) (Fig. 5). Of all the reported *Rph* genes, only *Rph20* on short arm of chromosome 5H was found within the proximity of the top 21 haplo-blocks. Three blocks (b001038, b001039 and b001040, each with a single marker) were mapped within 1 Mb distance from the *Rph20* locus. This proximity was further validated through BLAST searches of surrounding SNP marker loci of this study on four different genome assemblies (Supplementary Table 3).

To pinpoint the haplo-block for *Rph20* gene, LD patterns and blocking structures of SNP markers around the *Rph20* region were re-examined. Using progressively lower LD thresholds (starting from 0.1) and tolerance levels of two and three, marker loci 15–19 were identified within the same block with an LD threshold of 0.4 and tolerance two (Supplementary Table 4; Supplementary Fig. 8). This haplo-block is likely associated with *Rph20-*mediated resistance. Since maker loci 15 and 16 were found to be highly conserved and with very small SNP effect, only 17, 18 and 19 were considered as the representative marker loci for haplo-block *Rph20*. Further, allelic analysis tracing Australian barley cultivars for presence or absence of *Rph20* gene revealed that specific marker-allelic combination ‘*ATC* representing *Adenine, Guanine and Cytosine* bases’ at loci 17 (b001038), 18 (b001039) and 19 (b001040) (Supplementary Table 5). These markers were merged into a single haplo-block (designated as ‘*Rph20*’ in subsequent mentions) representing the known map location of *Rph20* on chromosome 5H.

### Harnessing haplotype diversity to achieve improved resistance

**Fig. 6:**
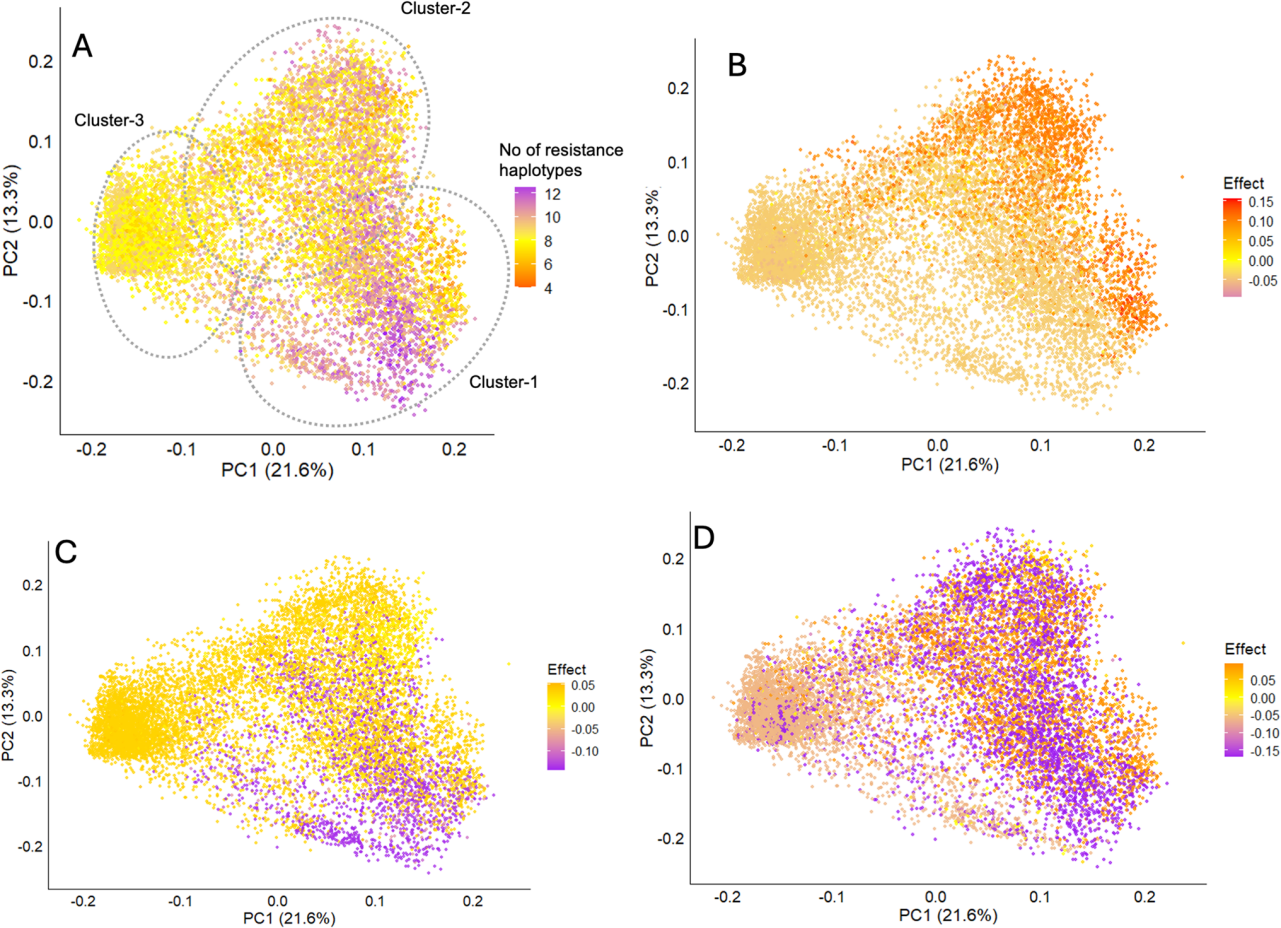
Distribution of resistance haplotypes within the population structure/germplasm pools across the breeding panel. Each dot in the scatter plot represents an individual genotype plotted against PC1 and PC2. **a** Genotypes colored according to the number of resistances haplo-blocks. **b** Genotypes colored for the block effect size for block b000305. **c** Genotypes colored for the block effect size for block b001125. **d** Genotypes colored for the block effect size for block Rph20

Plotting haplotype effects against block-wise scaled variance demonstrated high haplotypic diversity within the block and across the study panel. Analyzing resistance haplotypes within the context of the breeding population structure and selection history revealed that genotypes under clusters-1 and 2 exhibited more resistance haplotypes across the genome compared with the genotypes in the cluster-3 (Fig. 6a). Similarly, the haplo-blocks for stable resistance across varied environments (b000305, b001125 and *Rph20*) also showed higher haplotypic diversity for resistance effects in cluster-1 and 2 compared to the cluster-3 (Fig. 6b–d). For block b000305, the panel has 1282 unique haplotypes. 70% of the panel shared 465 unique haplotypes for resistance (effect < 0), while 0.5% genotypes shared 32 superior haplotypes for resistance (effect < − 0.07), of which 56 genotypes were in cluster-1 and cluster-2 and only nine genotypes in cluster 3. Similarly, for b001125, the panel has 725 unique haplotypes. 18% of the panel shared 336 unique haplotypes for resistance (effect < 0), while 16% genotypes shared superior haplotypes for resistance (effect < − 0.07), of which 1,907 genotypes (97%) under cluster-1 and cluster-2 and only 68 genotypes in cluster-3. Although this haplo-block confers many desirable haplotypes, genotypes under cluster-3 shared reduced diversity compared to other two clusters. For the *Rph20* haplo-block, seven unique allelic combinations were identified across the panel. However, 92% of the panel shared only three unique haplotypes. Five haplotypes contributed to the resistance (effect < 0) were present in 68% of the genotypes. The marker combination of ‘ATC’, identified previously as the superior haplotype, was shared by 30% of the total genotypes, predominantly (26%) from cluster-1 and cluster-2. These genotypes could be valuable sources of resistance haplotypes with higher effect size (−0.10) than the standard *Rph20* haplotype found in the majority of lines in cluster-3. The top 20 haplotypes for the haplo-blocks b000305 and b001125, along with their associated mean genotypic BLUEs, are presented in Supplementary Tables 7 and 8 and haplotypes of *Rph20* block in Supplementary Table 6.

Moreover, the linkage between the superior haplotypes for three stable blocks was also observed to narrow down the list of superior genotypes. Only 19 genotypes shared the highest effect haplotypes across all three: at b001125 (effect − 0.14), *Rph20* (effect − 0.17) and the haplotypes (with effect < − 0.04) at b000305. However, 575 genotypes (with average BLR score < 4) shared the same desirable haplotypes for b001125 and *Rph20* and a moderate effect haplotype (effect size − 0.036) at b000305.

**Fig. 7:**
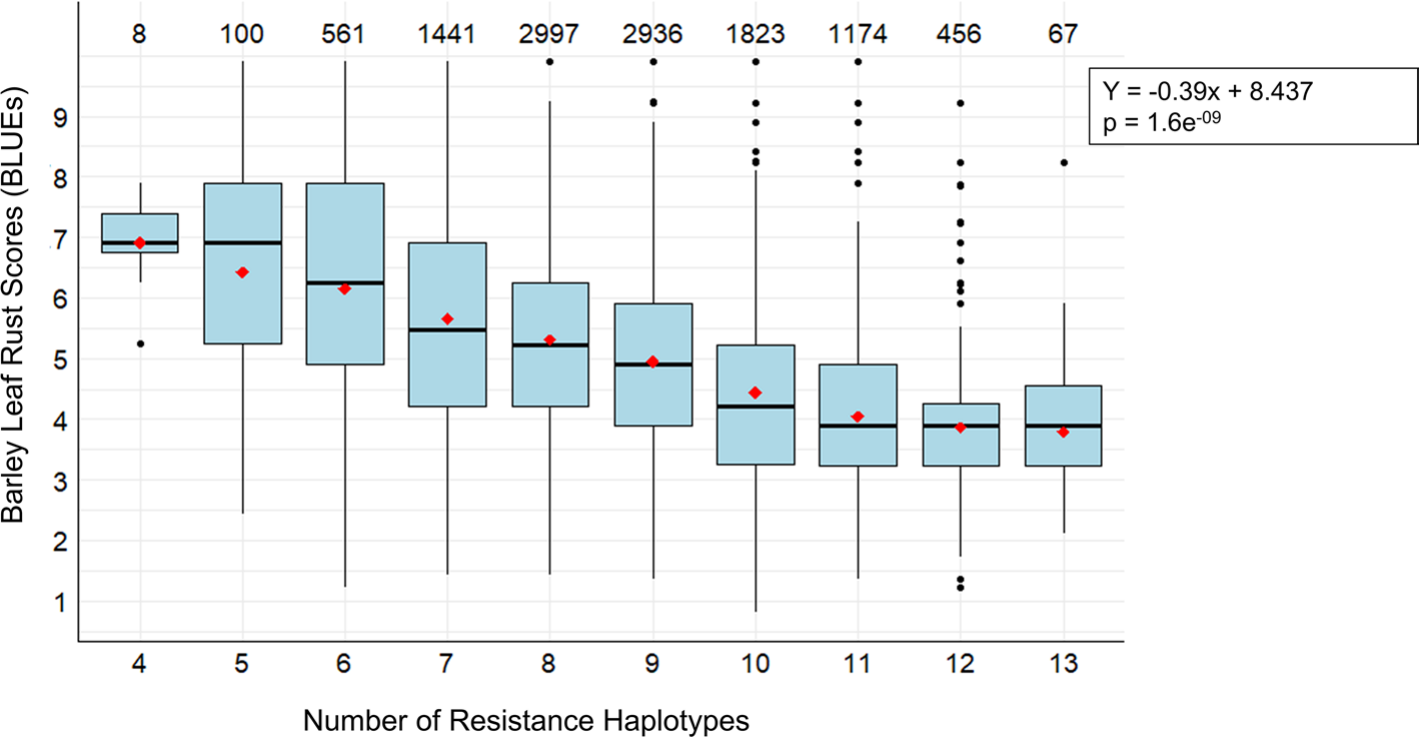
Stacking of resistance haplotypes plotted against their BLUEs from all environments (MET) analysis. The red dots inside the boxes represent mean BLUE values. The number at the top of each box plot represents the number of individuals within the group that contain the same number of resistant haplotypes stacked together

Stacking/pyramiding of the resistance haplotypes revealed a clear linear relationship between phenotypic score for resistance (BLUEs) and the number of favorable haplo-blocks per genotype (Fig. 7). Within the breeding panel, up to 13 resistance haplotypes were observed to be ‘stacked’ in 67 genotypes from the MET analysis. While additional resistance haplotypes beyond 11 did not further improve the mean rust score (BLUE), these genotypes exhibited greater stability in resistance, with lower variance in BLUE scores. Similar negative correlations were observed in other iClass-specific analyses as well for RMSD. Further, the relationship also holds true in the case of a group of 37 known commercial cultivars under MET condition (Supplementary Fig. 9).

## Discussion

### A diverse breeding panel for BLR characterization using LGEBV method

This study utilized a breeding panel developed and maintained by InterGrain Pty Ltd., in Australia. The genetically diverse panel was classified into three major clusters, originating from different crossing strategies and geographical origins, developed over multiple years at various breeding sites of InterGrain. A similar clustering pattern was reported by Brunner et al. (2024) in a smaller subset of the barley breeding panel and also by Hill et al. (2021b), who noted high genetic variation in Australian barley breeding populations, reflecting efforts to integrate diverse genetic backgrounds into breeding programs.

The breeding panel exhibited reasonably slow LD decay and in line with the expectations of a breeding population and thus resulted in the classification of haplo-blocks with an average of around three SNP markers. Strong directional selection, typical of commercial breeding, likely contributed to the pronounced LD patterns, leading to conserved haplotype blocks with lower recombination rates (Voss-Fels et al. 2019; Varshney et al. 2021). This pronounced LD facilitates the inheritance of favorable combinations of minor-effect alleles, collectively contributing to traits like rust resistance, a phenomenon known as ‘genetic hitchhiking’ (Bhat et al. 2021; Qian et al. 2016; Charlesworth et al. 2000; Turner-Hissong et al. 2020).

### Genotype-by-environment interactions in expression of BLR resistance

Substantial variability for BLR resistance at adult stage was observed across environments. This outcome is consistent with previous studies (Abdelghany et al. 2024; Ziems et al. 2017a; Singh et al. 2018; Ziems et al. 2023), highlighting the influence of environmental factors on APR expression in barley. We demonstrate significant cross-over GEI indicated by rank changes in genotype performance across the environment. Typically, BLR resistance is broadly considered to be environmentally stable; our finding of strong GEI challenges this assumption. While the literature studies lack such a comprehensive MET analysis for BLR resistance to explore GEI, stripe rust resistance in wheat has been studied recently (Vo Van-Zivkovic et al. 2025).

Partitioning GEI into clusters, like the iClass approach implemented in this study, provides a framework to manage this complexity. The analytical pipeline employed involved a two-step process effectively avoids potential ‘double shrinkage’ biases inherent in using BLUPs from random-effects models for downstream genetic association studies. A standard aspect of this widely used two-stage methodology, however, is that the uncertainty associated with the BLUE estimation is not explicitly carried forward into the subsequent mapping stage. Although this simplification could potentially influence the reported precision of haplotype effects, the approach remains robust for identifying and ranking major genetic effects across diverse overall conditions and within the specific environment types defined by iClass. A key consideration of the MET analysis implemented in this study is its latent nature, as the specific environmental and pathogenicity factors driving GEI remain unidentified. Emerging frameworks such as the integrated factor analytic linear mixed model (IFA-LMM; Tolhurst et al. 2021), which combine latent factor analysis with the environmental regressions, offer greater interpretability but rely on high-resolutions environmental data. Future studies should prioritize detailed environment and pathotype characterization to enable the application of such models for deeper insights into the GE networks underlying BLR resistance.

### Haplo-blocks associated with stability in BLR resistance

Across five LGEBV analysis, we detected haplo-blocks with high haplotype variance for resistance (Figs. 3, 5 and Supplementary Table 2), implying that substantial variation exists in Australian barley breeding panel for BLR resistance. The 21 most prominent haplo-blocks were of different sizes, number of haplotypes and their effects across different environmental conditions. Block b001093 was found to have the highest effect for BLR resistance in overall environments and in only one iClass PNN. However, as Gutierrez et al. (2015) highlighted, to ensure durability, resistance alleles must be present across multiple QTL, including both overall and environment-specific loci. Selecting top-performing haplo-blocks solely based on overall environmental performance may not ensure maximum effectiveness and durability of resistance. Durable resistance requires haplotypes to perform consistently across a wide range of environments, as well as under heavy disease pressure. So, for a block to be deemed environmentally stable, it must rank among the top blocks in all five haplotype analyses conducted in this study. Across five LGEBV analyses, five haplo-blocks (b000305, b001038, b001039, b001040 and b001125) consistently exhibited higher scaled variances above 0.4, suggesting that they are potential sources of environmentally stable BLR resistance and likely highly beneficial for breeding for the changing Australian environment (Fig. 3, Supplementary Table 2).

Of these five, three haplo-blocks (b001038, b001039 and b001040) were found comprising of a single SNP marker from the current set. These haplo-blocks were mapped in a very close proximity to the widely studied APR gene *Rph20*. A further exploration of this region was conducted with BLAST search using SNP data and published marker information (Supplementary Table 3) (Hickey et al. 2011; Dracatos et al. 2021; Ziems et al. 2023). Of these, marker in block b001039 was the primary driver for the higher resistance effect. Further analysis characterized the marker combination ‘*ATC’* with highest haplotype effect for *Rph20,* providing breeders with a high-resolution haplotype marker for better resistance. The detection of *Rph20* as one of the major multi-environment resistance factors was anticipated, given its long-standing presence in Australian barley germplasm. Its widespread integration into breeding lines has been enabled by Diversity Arrays Technology (DArT) and PCR-based markers, facilitating efficient selection and deployment (Dracatos et al. 2021; Hickey et al. 2012). Complementary APR genes such as *Rph23* and *Rph24* enhance the durability of *Rph20*-mediated resistance without compromising yield or grain quality (Ziems et al. 2017a; Singh et al. 2018), although limited marker development and field validation continue to hinder their broader utilization.

### Haplo-blocks for BLR resistance across environmental clusters

The study also identified high-variance haplo-blocks associated with BLR resistance for unique iClasses and across. Identifying environment adaptive QTL together with stable QTL are important for breeders to anticipate future disease pressures and design breeding strategies to develop resistant cultivars that balance durability and adaptability. For illustration purposes, here we discuss only about the haplo-blocks in iClass PPN. Haplo-block b000602, on the long arm of chromosome 3H, has the highest variance (one), potentially carrying QTL for resistance in specific environmental condition. Singly or a combination of disease pressure, pathotype accession and fluctuating environmental conditions may likely have caused phenotypic variation for resistance (Ziems et al. 2023; Rothwell et al. 2019). Some other studies have also reported variation in resistance expressions in barley to fluctuating temperatures and changing pathotypes (Norman et al. 2023; Singh et al. 2009, 2013; Hickey et al. 2011; Ziems et al. 2023). This block also includes the ortholog for *HvSgt1* gene, reported to regulate barley powdery mildew (*Mla*) resistance. So far, the linkage between BLR resistance and powdery mildew resistance has not been reported. However, *Sgt1* is a highly conserved regulatory components of disease resistance triggered by many R proteins (Bieri et al. 2004; Azevedo et al. 2002; Botër et al. 2007; Chapman et al. 2021). Additional haplo-blocks identified in iClass PPN include b000306 on 2H and b001521 and b001526 on 6H. The latter two haplo-blocks in 6H also harbor markers associated with net form net-blotch resistance (verified from the marker description provided by the developers of 40 K wheat–barley XT chips; Keeble-Gagnere et al. (2021)). Despite the lack of direct correlation between resistance to net blotch and BLR, conducting BLAST genome searches in the region resulted distinct resistance triggering kinase proteins (like serine/threonine protein kinase, nucleotide-binding leucine-rich repeat receptor (NLR) and Zinc-finger proteins) were co-located within the mapping distance of block b000306. The activation of an NLR by a fungal effector typically results in a host-resistance that prevents the growth of biotrophic and hemi-biotrophic pathogens (Balint-Kurti 2019a, b). However, necrotrophic pathogens can co-opt NLR effector recognition to promote cell death and enhance disease; with variable interactions across pathotype accessions (McCombe et al. 2022).

Similarly, block b001521 also harbors the *gpc-1* gene (for low grain protein content). Some studies have investigated the relationship between the *Gpc-B1* gene for high grain protein content in wheat and *Yr36* gene for stripe rust resistance (Gupta et al. 2022). But any linkage if lies between the *gpc-1* and leaf rust resistance could be the result of evolutionary mutation or the repulsion phase of the linkage. However, *gpc1* gene was a focus of European barley breeding programs (Distelfeld et al. 2008; Cai et al. 2013; Bertholdsson 1998) and Australian barley breeding populations have been reported strongly influenced by the European germplasm exchanges (Dracatos et al. 2021). Such co-location of multi-trait QTL possibly suggests selection hotspots for multiple traits in breeding programs. These findings suggest that there could be opportunities for breeders in emphasizing multi-trait selection. However, more evidence generated with development of accessible marker platforms and pan-genome information would potentially offer even further scope.

### Harnessing haplotype diversity to confer enhanced resistance

In the present study, accumulation of resistance haplotypes demonstrated a clear linear relationship with improved resistance in the study panel as well as in case of subset of commercial cultivars. Moreover, the relationship holds true at different environmental conditions as well as in overall environment to achieve stable resistance (Fig. 7), underscoring the highly quantitative nature of resistance. In addition, the extent of haplotype effect size and variance being variable across environmental conditions also suggests the quantitative nature of the trait and the genetic contribution of SNPs for resistance.

In the current breeding panel, nearly 80% of genotypes already exhibit 8–11 resistance haplotypes, along with 67 genotypes having as high as 13 resistance haplotypes for the mean BLR score below four. Here, the primary question arises, how do we achieve even better resistance? The existing haplotype diversity for resistance can be harnessed to enhance resistance through stacking favorable haplotypes. Which is practically achievable within a couple of years’ time by using cutting edge technologies like speed breeding and AI guided simulations to identify the best possible mating designs (Hayes et al. 2024). However, the existing linkage drag cannot be underestimated. As evidenced in the case of three stable blocks, no genotypes have the highest effect haplotypes for all the three haplo-blocks. Which possibly signifies the selection signatures observed toward the moderate effect haplotypes at b000305. That emphasizes the fact that informed breeding decisions could be made from the haplotype analysis to decide on selecting suitable haplotypes for stacking. Stacking favorable haplotypes not only improves BLR resistance but also ensures stability across environments, addressing the challenges of GEI.

Considering the local GEBV at haplo-block level can aid in designing intricate crosses between complementary genotypes, thereby enhancing breeding progeny with desirable haplotypes-termed as ‘ultimate genotype’ (Hayes et al. 2024). Future technologies like induced global and targeted recombination and synthetic biology to write desirable haplotypes may further support this approach (Bernardo 2017; Hayes et al. 2024). Similarly, integrating accelerated breeding cycles with haplotype-based genomic selection techniques could expedite the development and identification of superior recombinants, optimizing the use of existing genetic potential.

The retrospective use of high LD thresholds to perform LGEBV analysis in current breeding panel enabled the rapid identification of desirable haplo-blocks and their possible integration into breeding programs. This method will help bridge the gap between identifying genetic markers and practical breeding applications by facilitating genomics-driven crossing strategies. By fast-stacking haplotypes, breeders can capitalize on both known resistance genes like *Rph20* and other novel environment-specific resistance loci. By targeting key genomic regions and understanding their effects, breeders can achieve more durable BLR resistance outcomes, enhanced genetic gain and improved sustainability in barley breeding programs.

## Supplementary Information

Below is the link to the electronic supplementary material.

Supplementary file1 (DOCX 6588 kb)

## Data Availability

The data that support the findings of this study are available from InterGrain Pty Ltd, but restrictions apply to the availability of these data, which were used under license for the current study and so are not publicly available. Data are, however, available from the authors upon reasonable request and with permission of InterGrain Pty Ltd.
