# Supplementary material for: Exploring standing genetic variation for barley leaf rust resistance in Australian breeding panel
Source: Theor Appl Genet. 2026 Jan 11;139(1):31. doi: 10.1007/s00122-025-05122-4 (PMC12791067; doi:10.1007/s00122-025-05122-4)
Supplement: Supplementary file 1 — Supplementary file1 (DOCX 6588 kb) [file 122_2025_5122_MOESM1_ESM.docx]

**Supplementary materials**


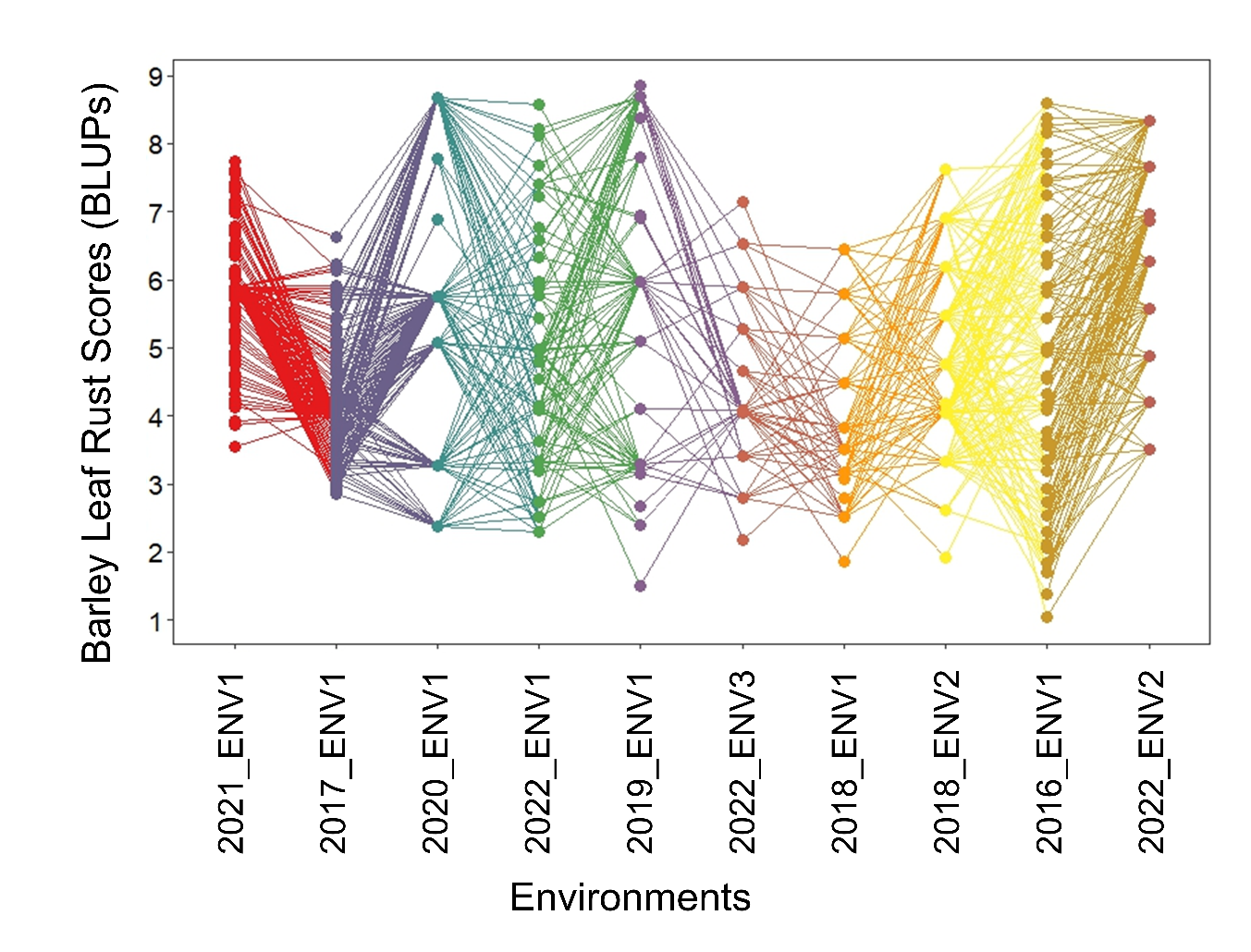


**Supplementary Fig.1**: BLUPs for leaf rust resistance scores in barley across ten study environments predicted using a Diag Model. X-axis: Environments (Year_location), Y-axis: Predicted Leaf Rust Score (BLUPs).


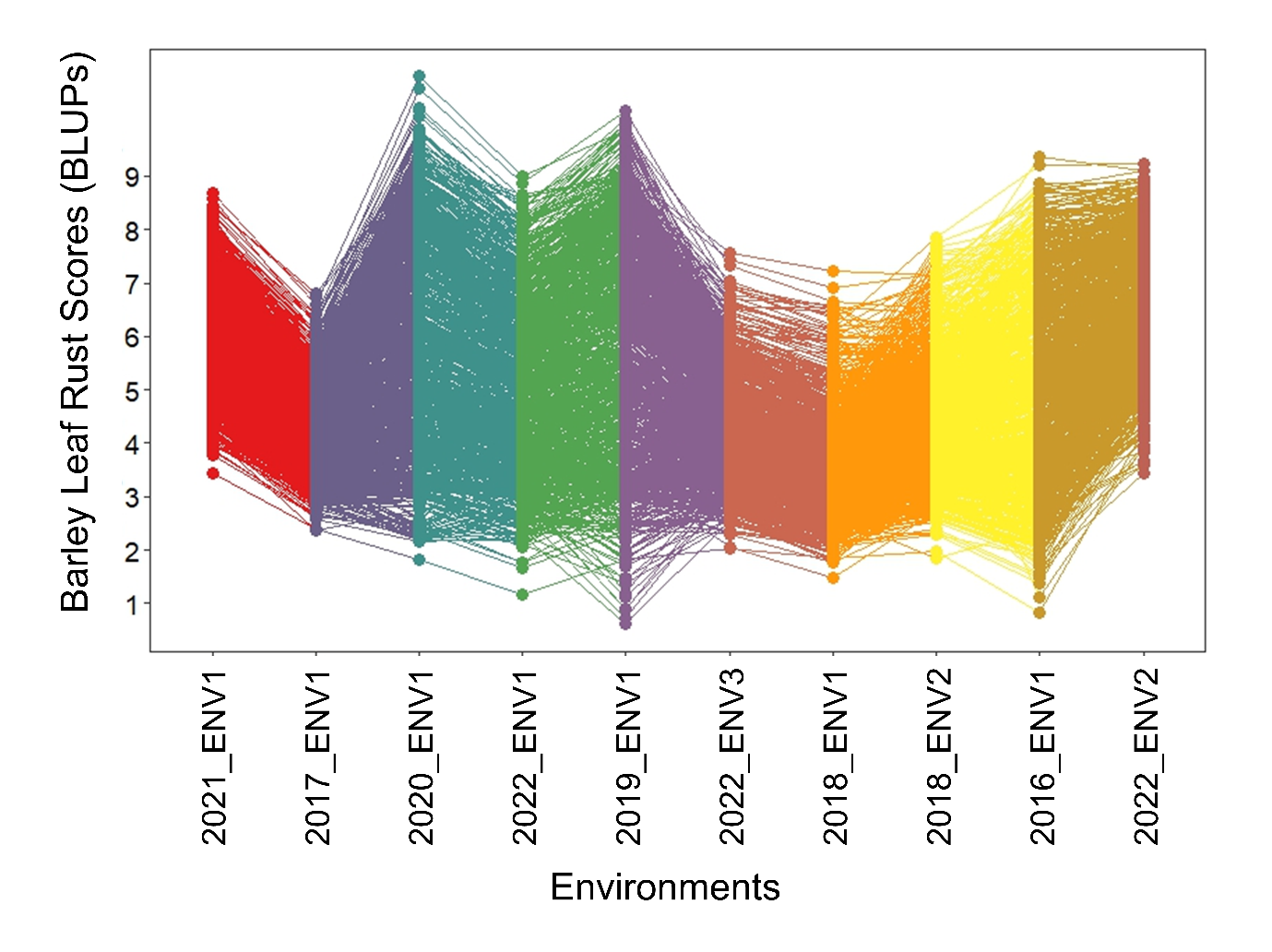


**Supplementary Fig.2**: Graphical visualisation of genotype by environment interactions for leaf rust score- best linear unbiased predictions (BLUPs) across 10 environments using factor analytic linear mixed model of the third order (FA3 model).


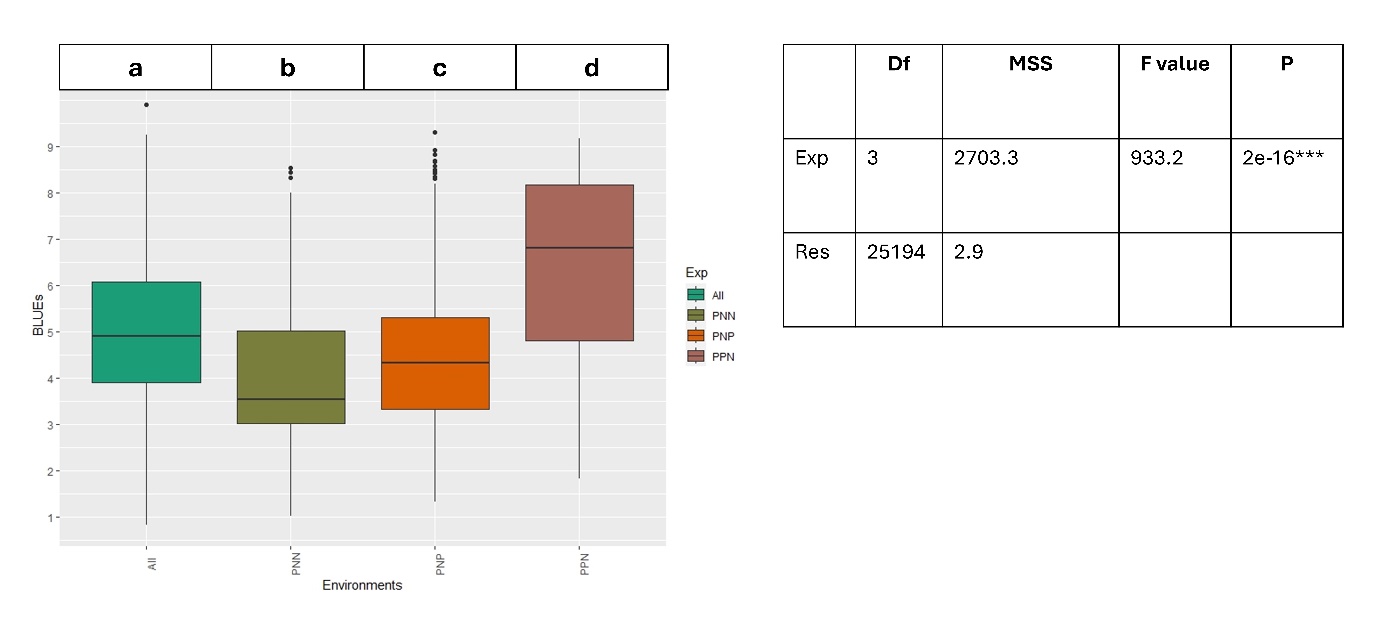


**Supplementary Fig.3**: Duncan’s multiple range test (DMRT) and comparison of mean BLUEs across ALL environments and iClasses PNN, PNP and PPN. ‘P’ and ‘N’ in iClasses refer to the positive and negative factor loadings for each factors from FA3 model.


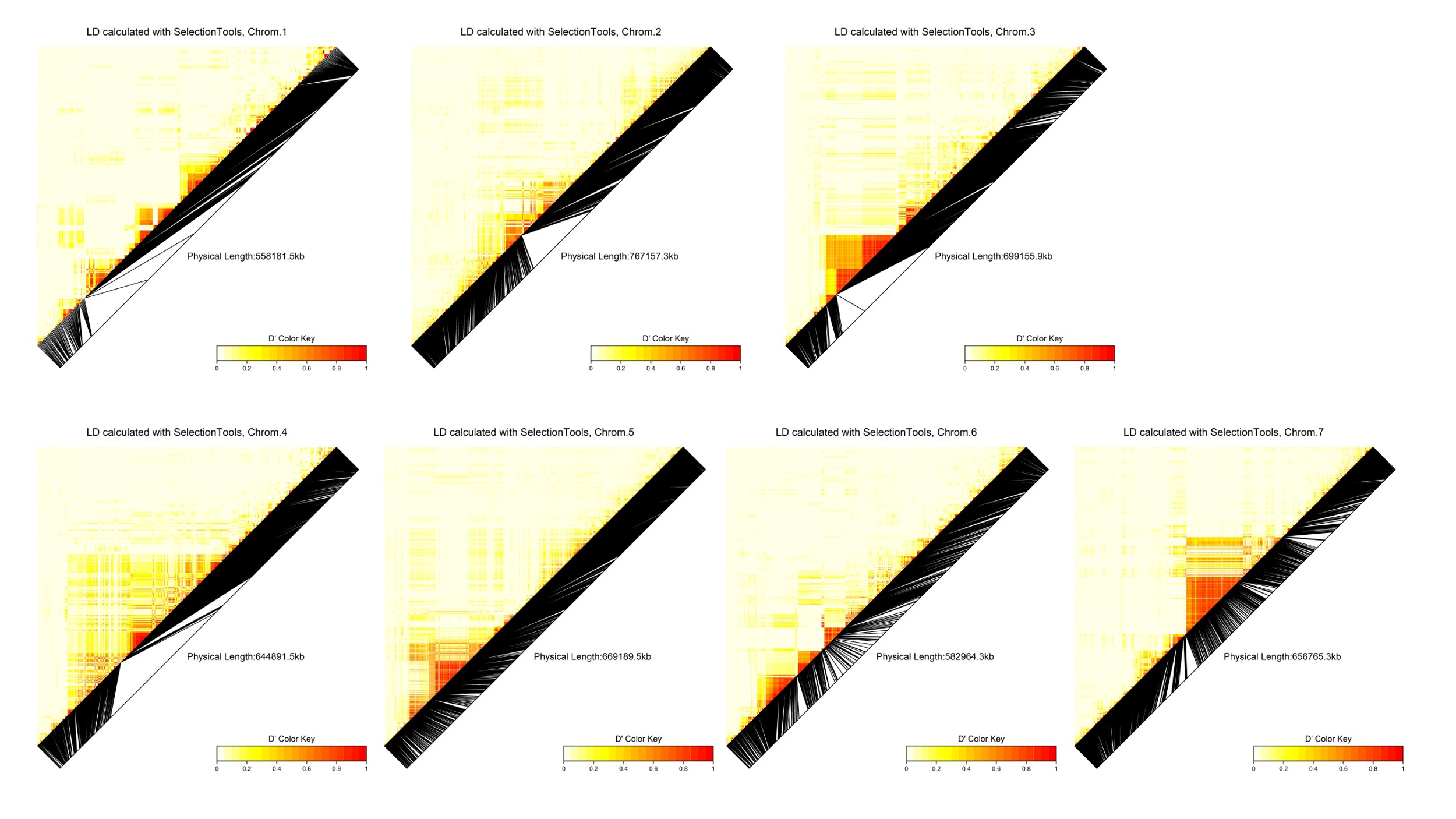
 **Supplementary Fig.4**: Heatmaps for pairwise linkage disequilibrium (LD) measured across seven chromosomes (1-7 from top-left to right)


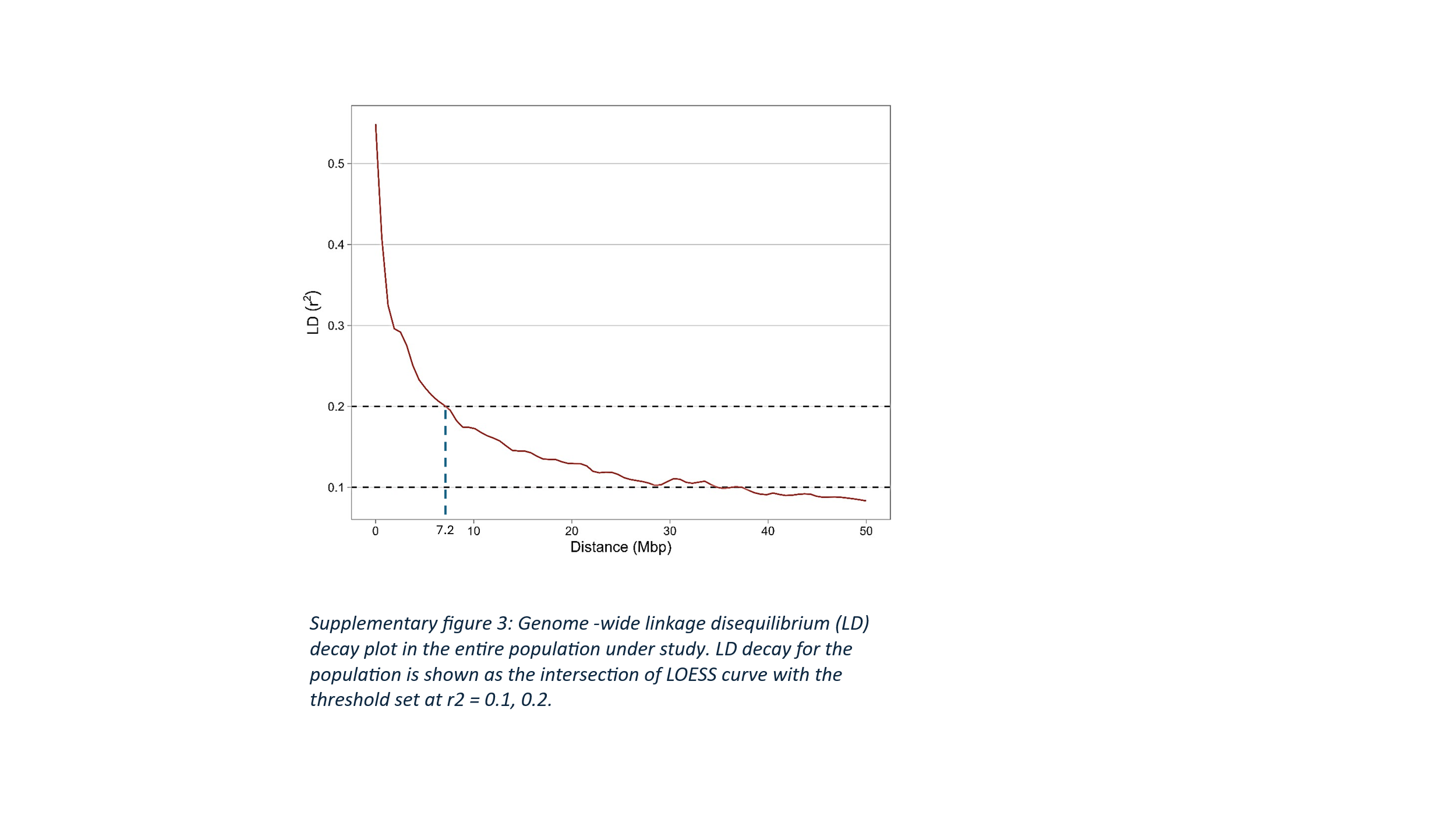


**Supplementary Fig.5**: Genome-wide LD decay plot in the entire population under study. LD decay for the population is shown as the intersection of LOESS curve with the threshold set at r^2^ = 0.1, 0.2.

**Supplementary Table 1**: Summary of LD blocks based on the LD threshold & tolerance parameters

| **LD** | **Tolerance** | **No of blocks** | **Avg no of markers/block** | **Remarks** |
| --- | --- | --- | --- | --- |
| 0.1 | 2 | 282 | 20.85 |  |
|  | 3 | 237 | 24.81 |  |
| 0.2 | 2 | 536 | 10.97 |  |
|  | 3 | 406 | 14.48 |  |
| 0.3 | 2 | 801 | 7.34 |  |
|  | 3 | 641 | 9.17 |  |
| 0.4 | 2 | 1090 | 5.39 |  |
|  | 3 | 926 | 6.35 |  |
| 0.5 | 2 | 1375 | 4.28 |  |
|  | 3 | 1191 | 4.94 |  |
| 0.6 | 2 | 1687 | 3.49 |  |
|  | 3 | 1464 | 4.02 |  |
| 0.7 | 2 | 2033 | 2.89 | Selected based on the LD decay and computational ease. |
|  | 3 | 1815 | 3.24 |  |
| 0.8 | 2 | 2646 | 2.22 |  |
|  | 3 | 2378 | 2.47 |  |


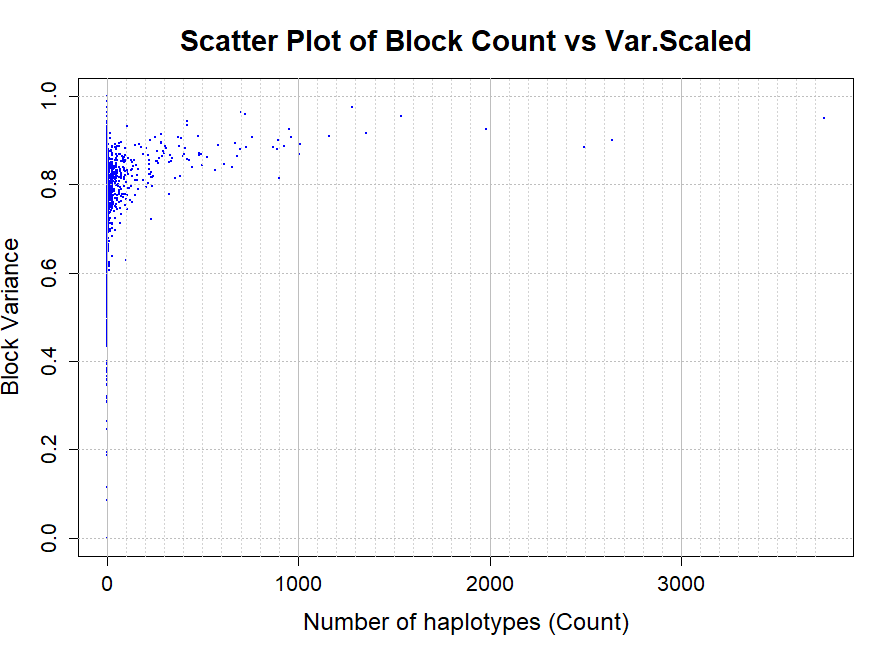
*
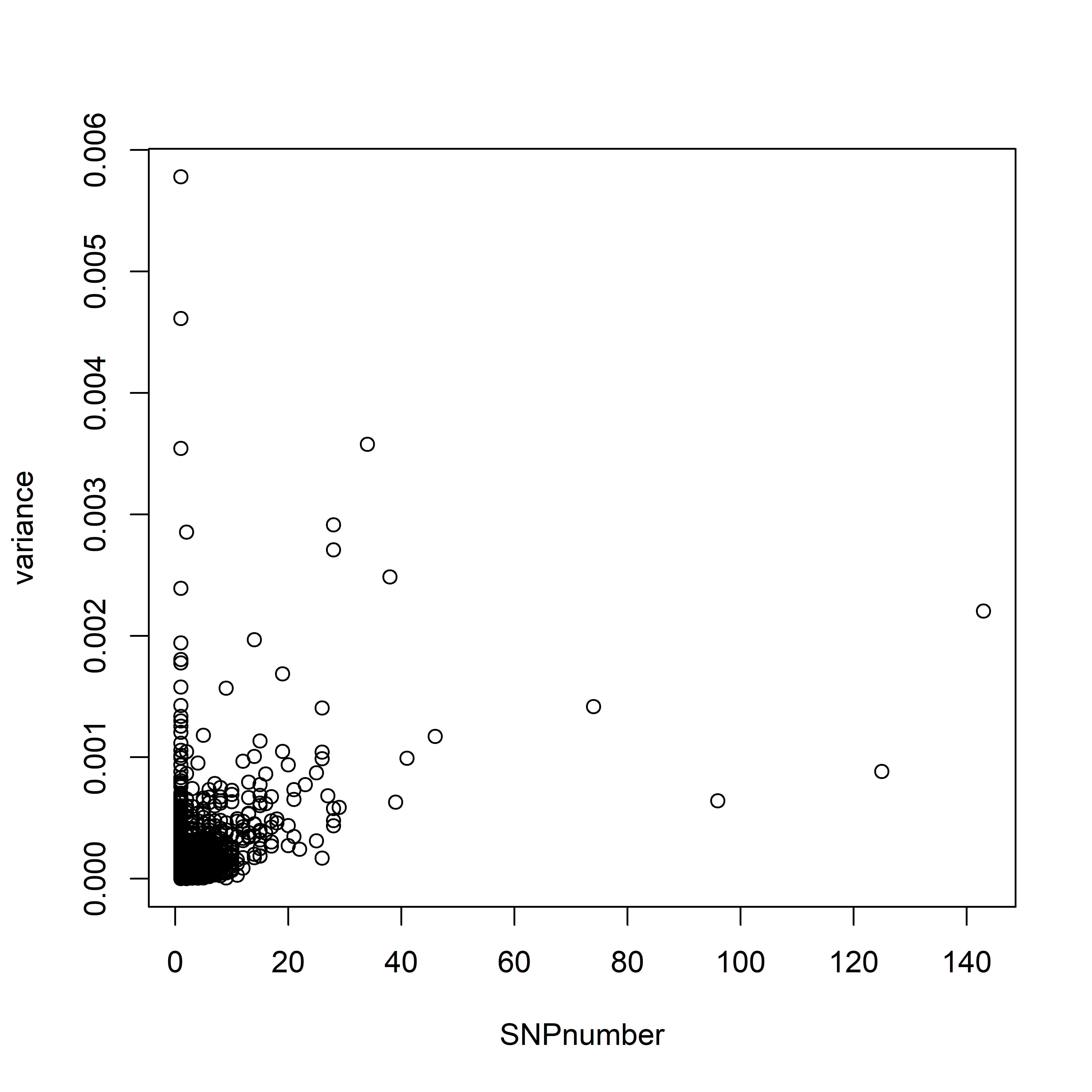
*

**Supplementary Fig.6**: Blocks variances of all 2033 haplo-blocks plotted against the number of haplotypes (A) and the respective SNP numbers (B).


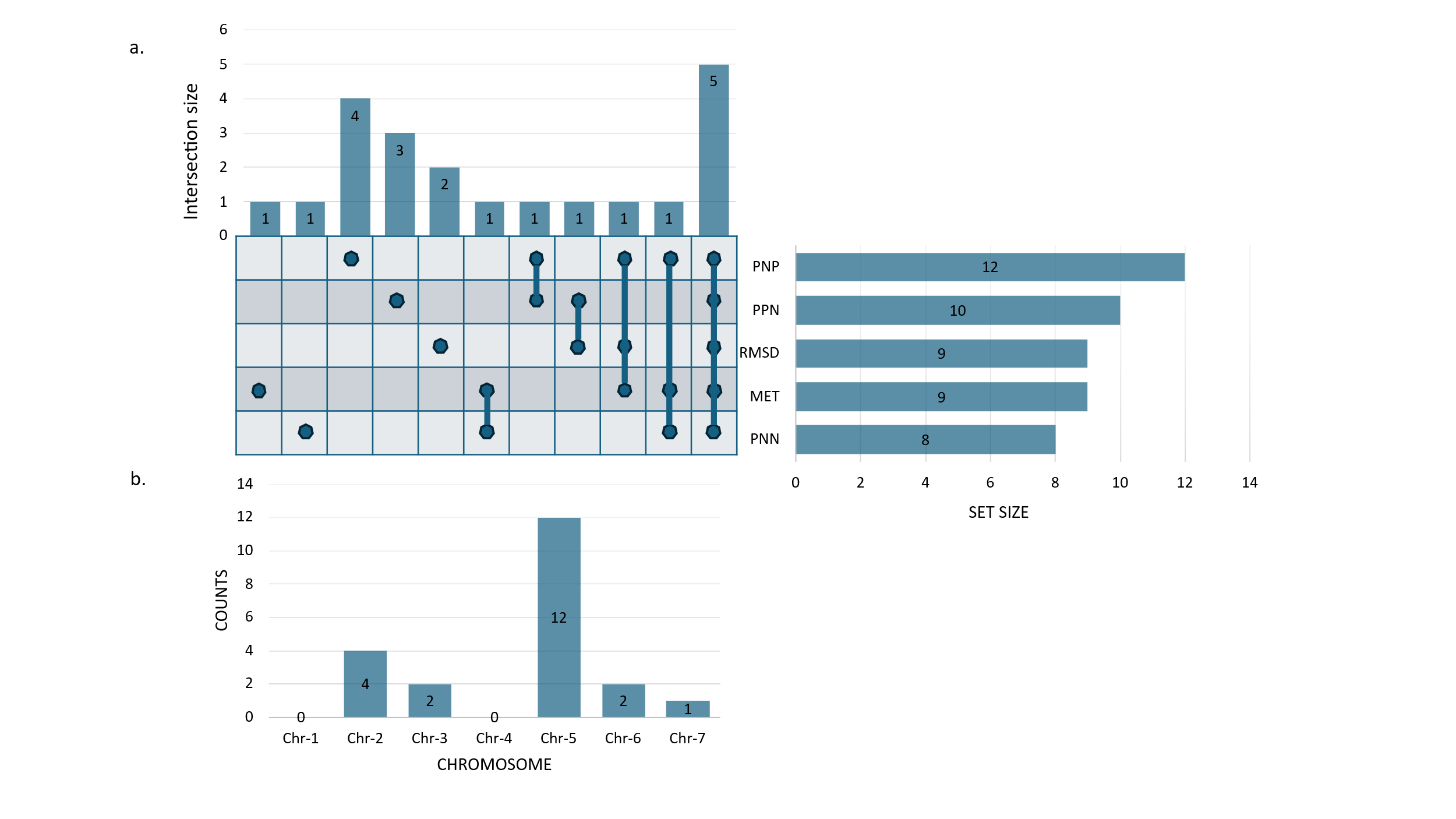


**Supplementary Fig.7**: An upset plot to show the intersection of high variance haplo-blocks (LD blocks) and their frequencies across the seven chromosomes. a) intersection across 5 analyses. b) frequency across chromosomes


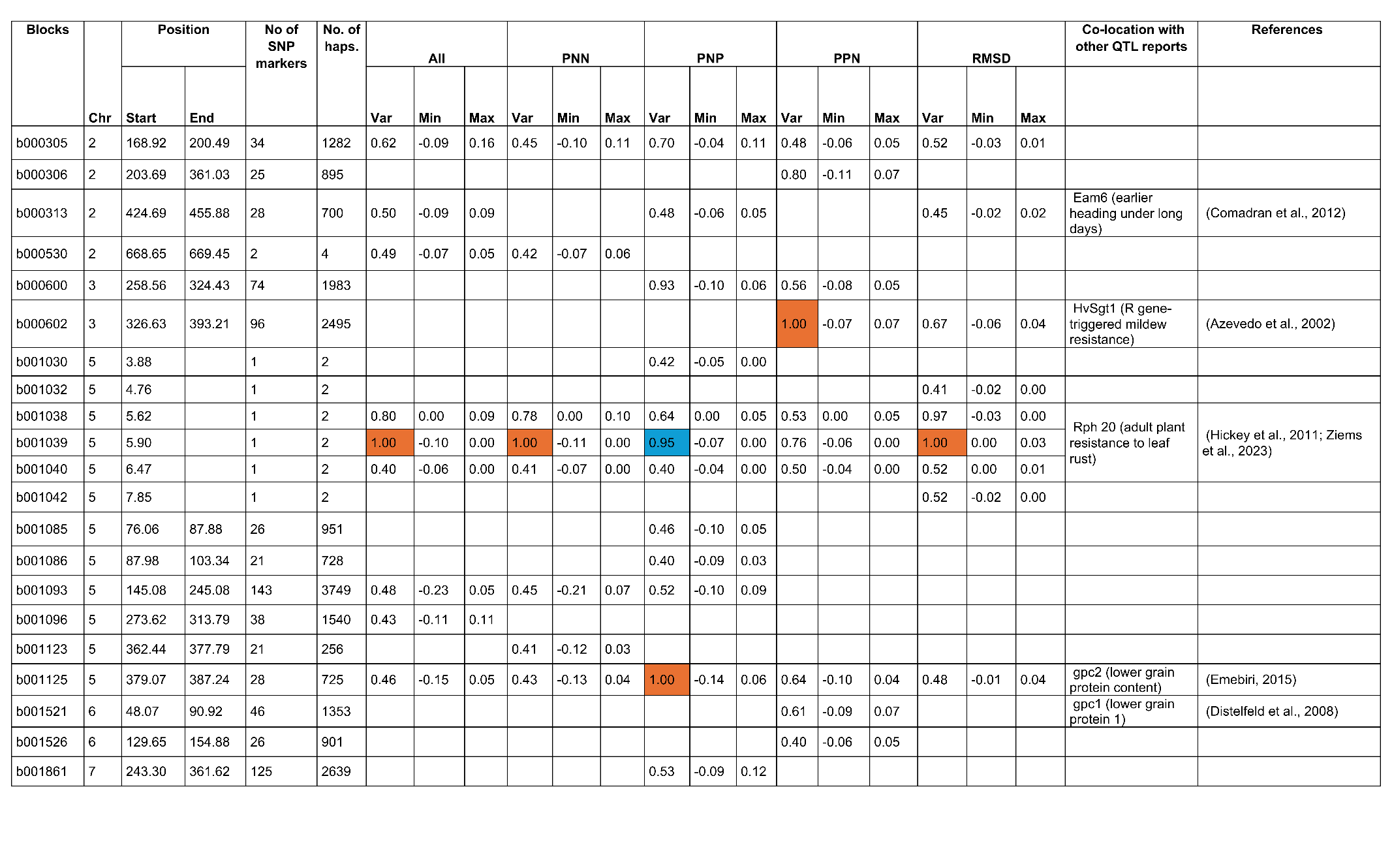


**Supplementary Table 2**: Significant haplo-blocks with their chromosomal (Chr) positions, variance and effect sizes in five different haplotype analysis (All-MET, iClasses-PNN, PNP and PPN, and RMSD). Highlighted rows represent blocks identified as significant across all the traits. Var: haplotype variance, E (Min): minimum haplotype effect & E (Max): maximum haplotype effect for respective block

**Supplementary Table 3**: BLAST results of marker loci (14-22) the block they constituted, sequences and physical positions (Mbp) together with known marker sequences and their positions for *Rph20* loci across multiple genome assemblies
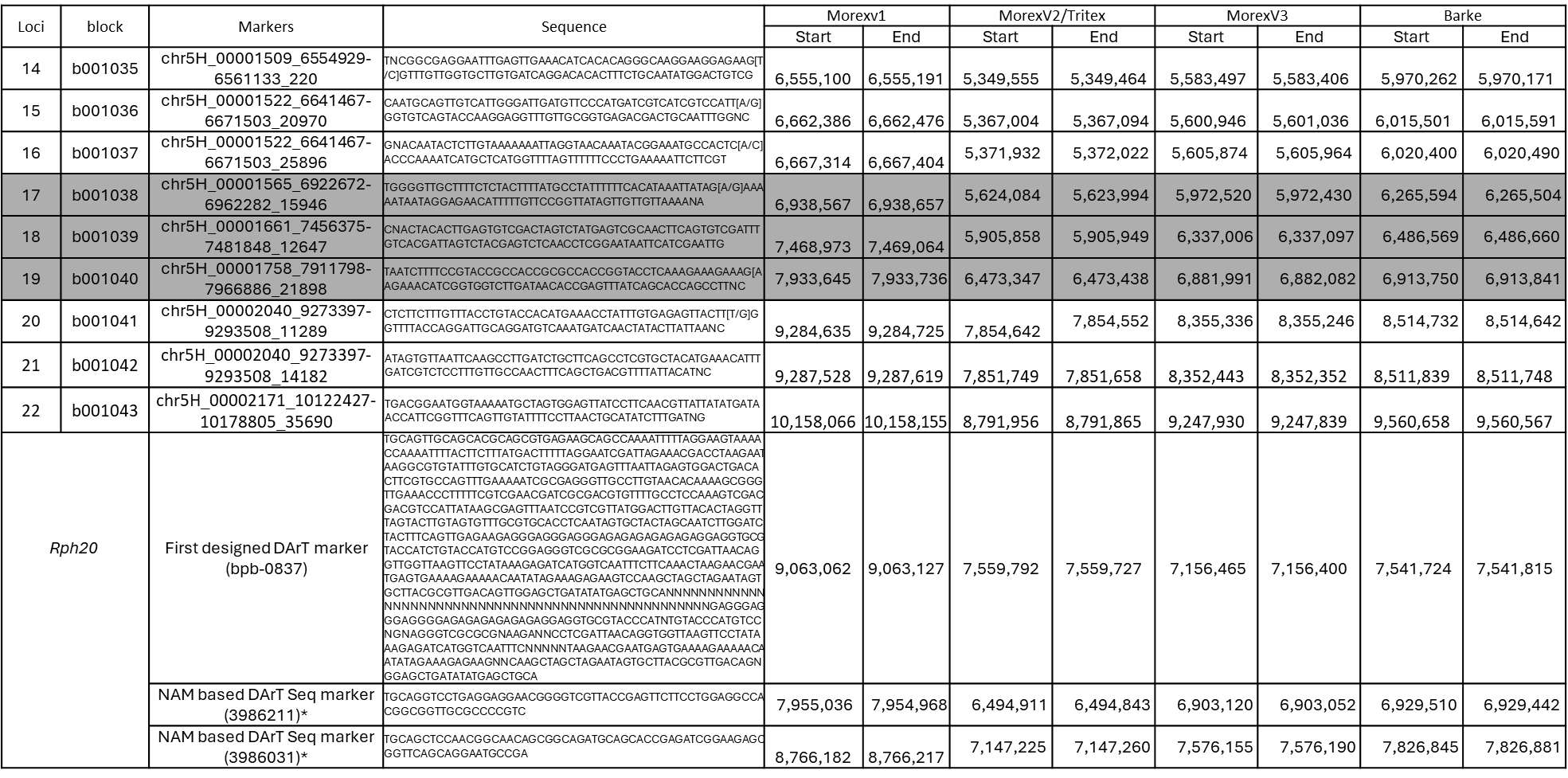


**Supplementary Table 4**: LD based blocking pattern of marker loci surrounding the position (Pos) of *Rph20* loci at differential combination of LD threshold and tolerance (T). The highlights are to track the marker loci representing blocks b001038=17, b001039=18 and b001040=19 in the current study. Note: the name of the block keeps changing with changed parameters


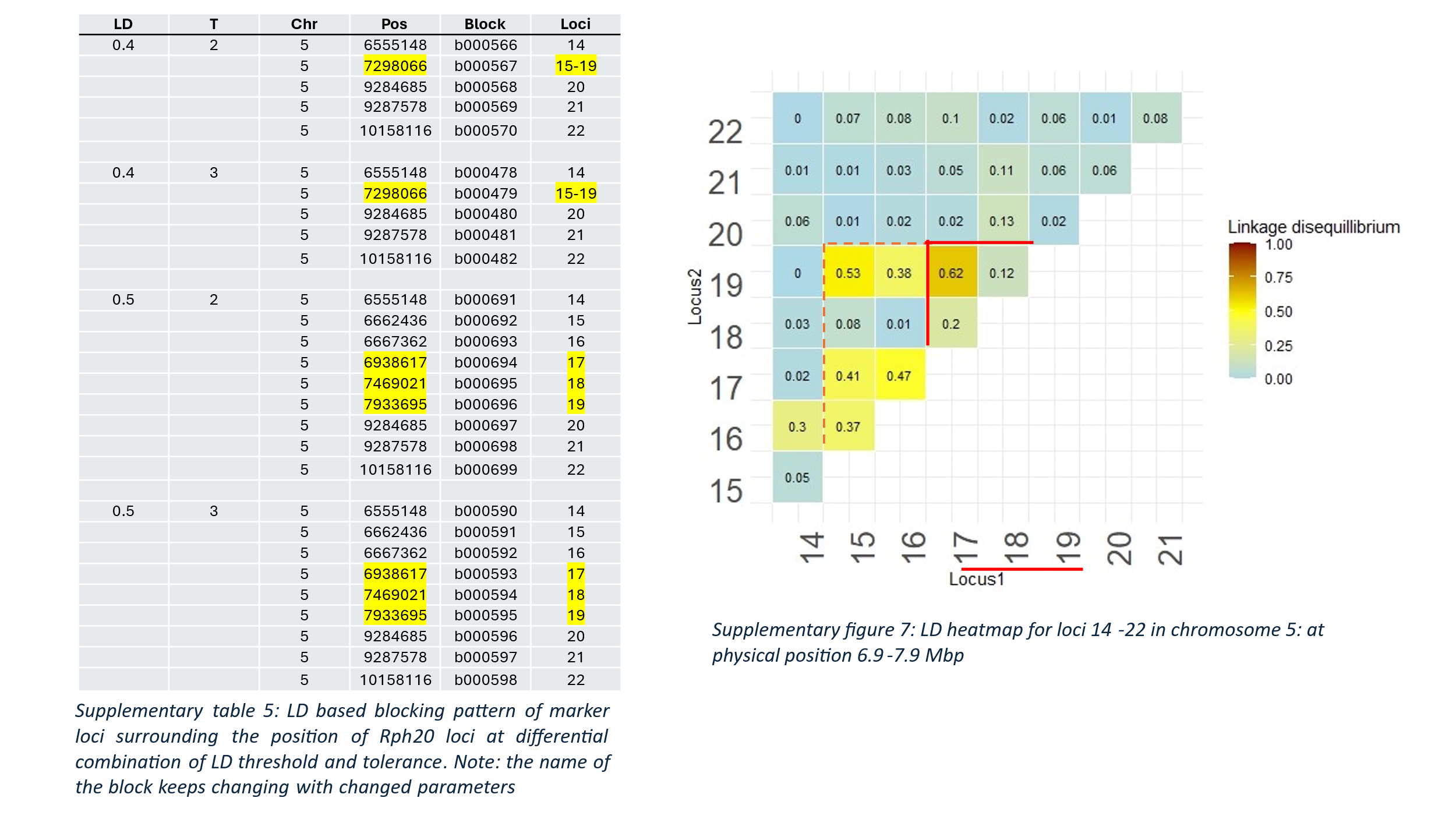

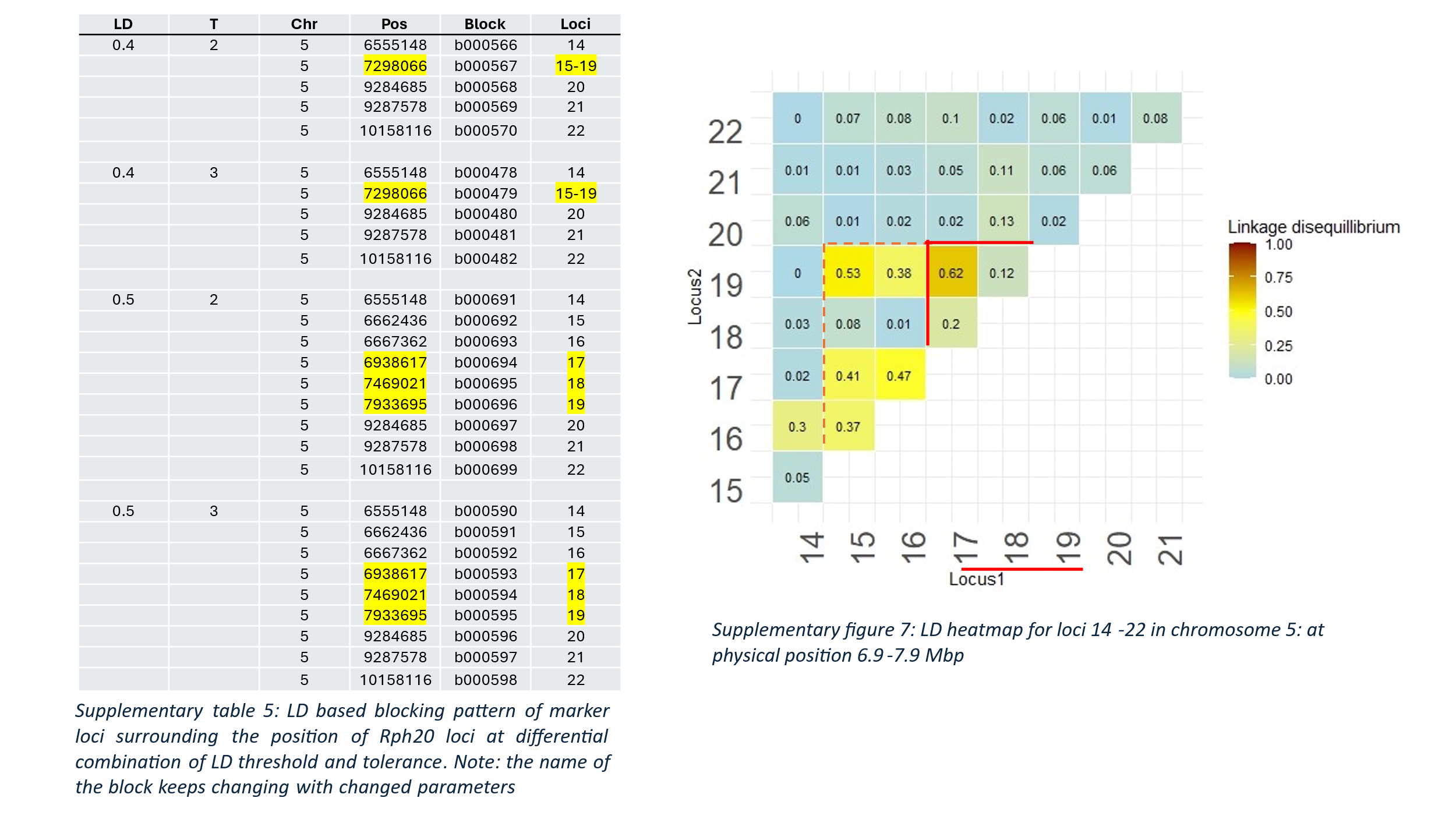


**Supplementary Fig.8**: LD heatmap for marker loci 14-22 in chromosome 5: at physical position 6.9-7.9 Mbp. The underscored loci 17, 18 and 19 representing b001038, b001039 and b001040.

**Supplementary Table 5**: Marker alleles around the *Rph20* region for Australian barley cultivars known for their presence (+)/absence (-) of *Rph20* and their phenotypic values (BLUE). The underscored loci 17, 18 and 19 representing blocks b001038, b001039 and b001040.

| **Genotypes** | **Loci→** | **10** | **11** | **12** | **13** | **14** | **15** | **16** | **17** | **18** | **19** | **20** | **(+/- *Rph20*)** |
| --- | --- | --- | --- | --- | --- | --- | --- | --- | --- | --- | --- | --- | --- |
|  | **BLUE↓** |  |  |  |  |  |  |  |  |  |  |  |  |
| Bottler | 3.1 | A | C | G | A | C | G | A | A | T | C | T | + |
| Dash | 2.4 | A | T | G | G | C | G | A | A | T | C | T | + |
| RGT-Planet | 3.5 | A | C | G | A | T | G | C | A | T | C | T | + |
| Rosalind | 1.6 | A | T | G | G | C | G | A | A | T | C | T | + |
| Granger | 3 | A | C | G | A | C | G | A | A | T | C | T | + |
| Oxford | 3.6 | A | C | G | A | C | G | A | A | T | C | T | + |
| Shepherd | 3 | A | C | G | A | C | G | A | A | T | C | T | + |
| Westminster | 2.7 | A | C | G | A | C | G | A | A | T | C | T | + |
| Hindmarsh | 4.6 | A | T | G | G | C | G | A | A | T | C | T | + |
| Flagship | 4.3 | A | C | G | A | C | G | A | A | T | C | T | + |
| Flinders | 3.7 | A | C | G | A | C | G | A | A | T | C | T | + |
|  | | | | | | | | | | | | | |
| Banks | 6.6 | A | T | G | G | C | A | C | G | C | A | T | - |
| Bass | 8.4 | A | T | G | G | C | A | C | G | C | A | T | - |
| Baudin | 7.8 | A | T | G | G | C | A | C | G | C | A | T | - |
| Lockyer | 4.42 | A | T | G | G | C | A | C | G | C | A | T | - |
| Compass | 8.3 | A | T | G | G | T | G | C | G | C | A | T | - |
| Navigator | 8.4 | A | T | G | G | C | A | C | G | C | A | T | - |
| Commander | 6.88 | A | T | G | G | C | A | C | G | C | A | T | - |
| VB9104 | 8.4 | A | T | G | G | C | A | C | G | C | A | T | - |
| Scope-CL | 6 | A | T | G | G | C | A | C | G | C | A | T | - |
| Buloke | 8.4 | A | T | G | G | C | A | C | G | C | A | T | - |
| Hamelin | 2.4 | A | T | G | G | C | A | C | G | C | A | T | - |
| Informative of *Rph20* | | No | Mix | No | Mix | Mix | Mix | Mix | Yes | Yes | Yes | No |  |

**Supplementary Table 6:** Haplotypes for block *Rph20* based on their effect sizes for resistance and corresponding details on mean rust score & genotype counts in the population. *: alternate/favourable allele with ≤ 0 effect size; (): Based on a sample study of 20 genotypes with their known presence (+)/absence (-) of *Rph20* gene. The underscored loci 17, 18 and 19 represent haplo-blocks b001038, b001039 and b001040.

| **Effect** | **Mean_BLR** | **Genotype**  **count** | **Marker alleles for block *Rph20*** | | | **(+/-) *Rph20* gene** |
| --- | --- | --- | --- | --- | --- | --- |
|  |  |  | **17** | **18** | **19** |  |
| -0.168 | 4.21 | 3620 | A* | T* | C* | (+) |
| -0.108 | 5.43 | 154 | A* | T* | A | N/A |
| -0.072 | 4.89 | 54 | G | T* | C* | N/A |
| -0.060 | 5.01 | 4198 | A* | C | C* | N/A |
| 0.000 | 4.38 | 97 | A* | C | A | N/A |
| 0.036 | 5.91 | 660 | G | C | C* | (-) |
| 0.096 | 5.71 | 2780 | G | C | A | (-) |

| **Effect** | **Mean_BLR** | **Genotype**  **count** | **Marker alleles for block b000305** | | | | | | | | | | | | | | | | | | | | | | | | | | | | | | | | | |
| --- | --- | --- | --- | --- | --- | --- | --- | --- | --- | --- | --- | --- | --- | --- | --- | --- | --- | --- | --- | --- | --- | --- | --- | --- | --- | --- | --- | --- | --- | --- | --- | --- | --- | --- | --- | --- |
| -0.086 | 4.84 | 13 | A | A | G | G | A | A | G | T | C | T | C | C | C | A | C | A | G | T | C | G | G | C | A | A | A | A | T | C | G | A | C | A | A | G |
| -0.076 | 4.82 | 15 | A | A | G | G | A | A | G | T | C | T | C | C | C | A | C | A | G | T | C | G | G | T | A | A | A | A | T | C | G | A | C | A | A | G |
| -0.070 | 5.18 | 19 | A | A | G | G | A | A | G | T | C | T | T | C | C | A | C | A | G | T | C | A | G | C | A | A | A | A | T | C | G | A | C | A | A | G |
| -0.057 | 4.79 | 37 | A | A | G | G | A | A | G | T | C | T | C | C | C | A | C | A | G | T | C | G | G | T | A | A | A | A | T | C | G | A | C | C | A | G |
| -0.054 | 4.05 | 116 | A | A | G | G | A | A | G | T | C | T | C | C | C | A | C | G | G | T | C | G | G | T | A | A | A | G | T | C | G | A | C | C | A | G |
| -0.050 | 4.83 | 31 | A | A | G | G | A | A | G | T | C | T | C | C | C | A | C | A | G | T | C | G | G | C | A | A | A | G | T | C | G | A | C | C | A | G |
| -0.045 | 4.76 | 18 | A | A | G | G | A | A | G | C | C | T | C | T | C | A | C | G | A | G | C | G | A | T | G | G | G | A | C | T | A | G | A | A | G | G |
| -0.040 | 4.88 | 45 | A | A | G | G | A | A | G | T | C | T | C | C | C | A | C | A | G | T | C | G | G | T | A | A | G | G | T | C | G | A | C | C | A | G |
| -0.040 | 4.91 | 7187 | A | A | G | G | A | A | G | T | C | T | C | C | C | A | C | A | G | T | C | G | G | T | A | A | A | G | T | C | G | A | C | C | A | G |
| -0.038 | 5.20 | 30 | A | A | G | G | A | A | G | T | C | T | T | C | C | C | C | G | G | T | C | G | G | T | A | A | A | G | T | C | G | A | C | C | A | G |
| -0.036 | 5.16 | 35 | A | A | G | G | A | A | G | T | C | T | C | C | C | A | C | A | G | T | C | G | G | T | A | A | A | G | T | C | G | A | C | C | A | A |
| -0.035 | 4.74 | 29 | A | A | G | G | A | A | G | T | C | T | T | C | C | C | C | G | G | T | C | G | G | T | A | A | A | G | T | C | G | A | C | C | A | A |
| -0.033 | 5.62 | 24 | A | A | G | G | A | A | G | T | C | T | C | C | C | A | C | A | G | T | C | G | G | T | A | A | A | G | T | C | A | A | C | C | A | G |
| -0.032 | 5.19 | 87 | A | A | G | G | A | A | G | T | C | T | C | C | C | A | C | A | G | T | C | G | G | T | A | G | A | G | T | C | G | A | C | C | A | G |
| -0.032 | 5.08 | 68 | A | A | G | G | A | A | G | T | C | T | C | C | C | A | C | A | G | T | C | G | G | T | A | A | A | G | C | C | G | A | C | C | A | G |
| -0.030 | 4.97 | 21 | A | A | G | G | A | A | G | T | C | T | C | C | C | A | C | A | G | T | C | G | G | T | A | A | A | G | T | C | A | A | C | C | A | A |
| -0.024 | 5.36 | 29 | A | A | G | G | A | A | G | T | C | T | T | C | C | A | C | A | G | T | C | G | G | T | A | A | A | G | T | C | G | A | C | C | A | G |
| -0.023 | 5.38 | 54 | A | A | G | G | A | A | G | T | C | T | C | T | C | A | C | A | G | T | C | G | G | T | A | A | A | G | T | C | G | A | C | C | A | G |
| -0.022 | 4.63 | 11 | A | A | G | G | A | A | G | T | C | T | C | C | C | A | C | A | G | T | C | G | G | T | A | A | A | G | C | C | A | A | C | C | A | A |
| -0.020 | 5.29 | 12 | A | A | G | G | A | A | A | T | C | T | C | C | C | A | C | A | G | T | C | G | G | T | A | A | A | G | T | C | G | A | C | C | A | G |

**Supplementary Table 7**: Top 20 haplotypes for block b000305 based on their effect sizes for resistance and corresponding details on mean rust score & genotype counts in the population

| **Effect** | **Mean_BLR** | **Genotype**  **count** | **Marker alleles for block b001125** | | | | | | | | | | | | | | | | | | | | | | | | | | | |
| --- | --- | --- | --- | --- | --- | --- | --- | --- | --- | --- | --- | --- | --- | --- | --- | --- | --- | --- | --- | --- | --- | --- | --- | --- | --- | --- | --- | --- | --- | --- |
| -0.138 | 4.42 | 1503 | T | G | A | G | C | G | T | G | C | C | C | A | T | G | C | G | A | G | G | G | C | A | G | C | T | A | C | T |
| -0.137 | 5.60 | 5 | T | G | A | G | C | G | T | G | C | C | C | A | T | G | C | G | A | G | G | A | C | A | G | C | T | A | C | T |
| -0.129 | 3.60 | 7 | T | G | A | G | C | G | T | G | C | C | C | A | T | G | C | G | A | A | G | G | C | A | G | C | T | A | C | T |
| -0.128 | 5.25 | 5 | T | A | A | G | C | G | T | G | C | C | C | A | T | G | C | G | A | G | G | G | C | A | G | C | T | A | C | T |
| -0.128 | 5.21 | 32 | T | G | A | G | C | G | T | G | C | C | C | A | T | G | C | G | A | G | G | G | C | A | G | C | T | A | C | G |
| -0.123 | 4.63 | 90 | T | G | A | G | C | G | T | G | C | C | C | A | T | G | C | G | A | G | G | G | A | A | G | C | T | A | C | T |
| -0.113 | 4.78 | 18 | T | G | A | G | C | A | T | G | C | C | C | A | T | G | C | G | A | G | G | G | C | A | G | C | T | A | C | T |
| -0.112 | 3.80 | 10 | T | G | A | G | C | G | T | G | C | C | C | A | T | G | C | G | A | G | G | G | C | A | A | C | T | A | C | T |
| -0.102 | 3.56 | 14 | T | G | A | G | C | A | T | G | C | C | C | A | T | G | C | G | A | G | G | G | C | A | G | C | T | A | C | G |
| -0.087 | 4.74 | 22 | T | G | A | G | C | A | T | G | C | C | C | A | T | G | C | G | A | G | G | G | C | A | A | C | T | A | C | T |
| -0.078 | 4.68 | 41 | T | G | A | G | C | A | T | G | C | C | C | A | T | G | C | G | A | G | G | G | C | A | A | C | T | A | T | G |
| -0.076 | 4.58 | 137 | T | G | A | G | C | A | T | G | C | C | C | A | T | G | C | G | A | G | G | G | C | A | A | C | T | A | C | G |
| -0.069 | 4.39 | 12 | T | G | A | G | C | A | T | G | C | C | C | A | T | G | C | G | A | A | G | G | C | A | A | C | T | A | T | G |
| -0.067 | 4.45 | 9 | T | G | A | G | C | A | T | G | C | C | C | A | T | G | C | G | A | A | G | G | C | A | A | C | T | A | C | G |
| -0.066 | 4.56 | 13 | T | G | A | G | C | A | T | G | C | T | C | A | T | G | C | G | A | G | G | G | C | A | A | C | T | A | C | G |
| -0.029 | 4.75 | 6 | G | A | A | A | C | G | T | T | A | T | C | G | C | A | A | G | A | A | T | G | A | C | G | C | C | G | C | T |
| -0.028 | 4.62 | 8 | G | A | A | A | C | G | T | T | A | T | C | G | C | A | A | G | A | A | T | A | A | C | G | C | C | G | C | T |
| -0.012 | 3.26 | 12 | G | G | A | A | C | A | T | G | C | T | A | A | C | A | A | G | A | A | T | G | A | C | A | T | T | G | C | G |
| -0.008 | 4.27 | 13 | G | G | A | A | C | A | C | G | A | T | A | A | C | A | C | A | G | A | T | G | A | C | A | T | T | G | T | G |
| -0.002 | 6.30 | 15 | G | G | A | A | C | A | C | G | A | T | A | A | C | A | C | A | G | A | T | G | A | C | A | C | C | G | C | G |

**Supplementary Table 8**: Top 20 haplotypes for block b001125 based on their effect sizes for resistance and corresponding details on mean rust score & genotype counts in the population


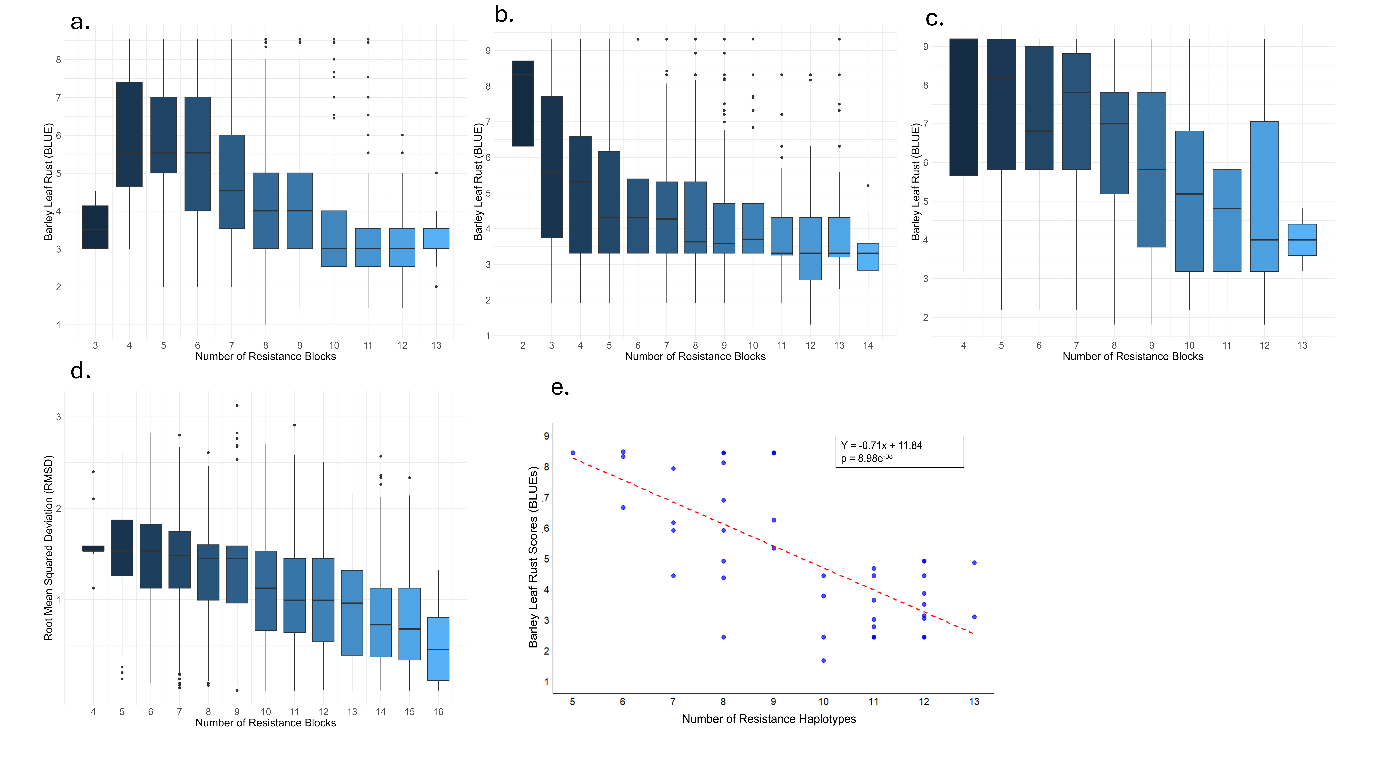


**Supplementary Fig.9**: Stacking of resistance haplotypes plotted against their BLUEs from (a) PNN (b) PNP (c) PPN (d) RMSD and (e) known commercial varieties under MET analysis.


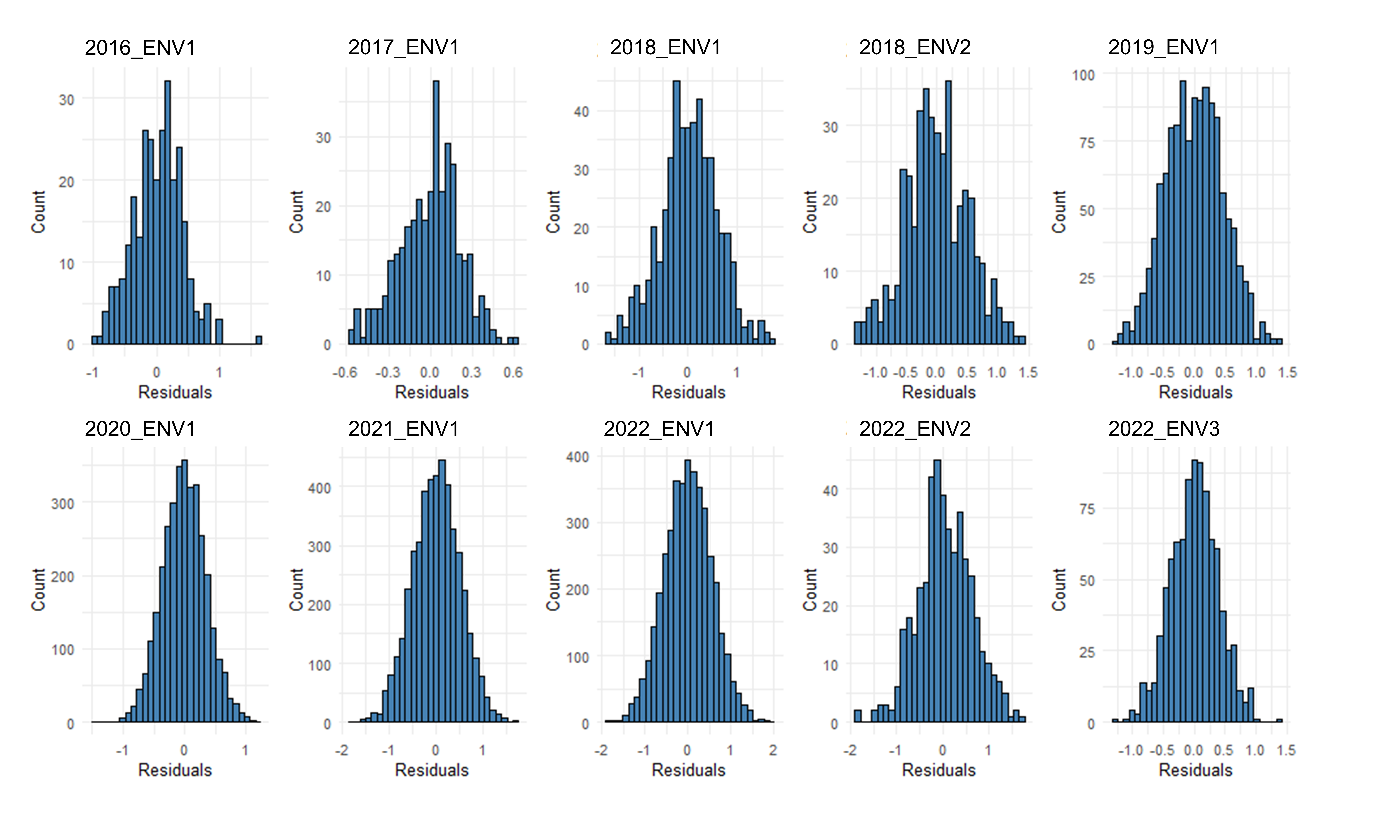


**Supplementary Information**: Histogram of residuals for barley leaf rust scores across ten environments.
